# Expression of heterologous sigma factors enables functional screening of metagenomic and heterologous genomic libraries

**DOI:** 10.1038/ncomms8045

**Published:** 2015-05-06

**Authors:** Stefan M. Gaida, Nicholas R. Sandoval, Sergios A. Nicolaou, Yili Chen, Keerthi P. Venkataramanan, Eleftherios T. Papoutsakis

**Affiliations:** 1 Department of Chemical and Biomolecular Engineering, Molecular Biotechnology Laboratory, Delaware Biotechnology Institute, University of Delaware, 15 Innovation Way, Newark, Delaware 19711, USA

## Abstract

A key limitation in using heterologous genomic or metagenomic libraries in functional genomics and genome engineering is the low expression of heterologous genes in screening hosts, such as *Escherichia coli*. To overcome this limitation, here we generate *E. coli* strains capable of recognizing heterologous promoters by expressing heterologous sigma factors. Among seven sigma factors tested, RpoD from *Lactobacillus plantarum* (*Lpl*) appears to be able of initiating transcription from all sources of DNA. Using the promoter GFP-trap concept, we successfully screen several heterologous and metagenomic DNA libraries, thus enlarging the genomic space that can be functionally sampled in *E. coli*. For an application, we show that screening fosmid-based *Lpl* genomic libraries in an *E. coli* strain with a chromosomally integrated *Lpl rpoD* enables the identification of *Lpl* genetic determinants imparting strong ethanol tolerance in *E. coli*. Transcriptome analysis confirms increased expression of heterologous genes in the engineered strain.

## Abstract (web summary)

Screening genomic or metagenomic libraries for interesting products or activities is often hampered by poor gene expression in a heterologous host. Here the authors show that the expression of a *Lactobacillus* sigma factor greatly enhances transcription of heterologous and environmental DNA in *E. coli*.

## Graphical abstract

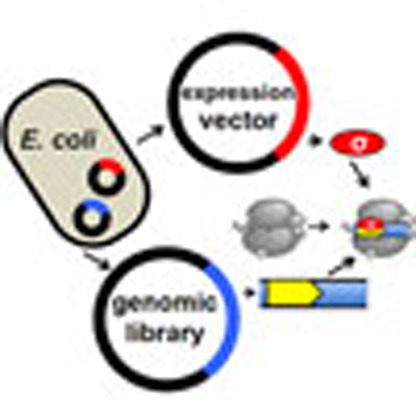

It is widely recognized^1^ that the microbial diversity present in nature^2^,^3^, coupled with fast evolution rates^4^, results in an enormous stock of genetic material that remains largely unexplored for either fundamental studies or biotechnological applications^5^. With fewer than 1% of the organisms having being successfully cultured in the laboratory so far^1^, this enormous genetic diversity can be harvested best in the form of metagenomic libraries^1^,^3^,^6^. Effective functional screening of metagenomic libraries can unlock the hidden potential of the genetic diversity in nature and lead to the identification of novel or potent enzymatic activities as well as cellular programs, which could be used to engineer superior strains for biotechnological applications^7^. Effective screening of metagenomic libraries ‘is grossly limited by the ability of the organism that is hosting the metagenomic library to express genes from anonymous organisms represented in the library'^1^. This is largely attributed to the inability of the transcriptional machinery of the host organism to recognize promoters from the metagenome and possibly also translate metagenomic transcripts^1^. A similar situation prevents expansion of the genomic space that can be explored in the context of genome engineering^8^,^9^,^10^, whereby, so far, the genomic diversity that can be generated and screened for developing useful strains is confined to mutational perturbations of single genomes^11^,^12^,^13^. As a result, novel genes, genetic programs or pathways outside single genomes are largely not accessible.

A larger functional sample space (defined as the fraction of expressed genes from a given genomic space) is a prerequisite for successful activity-based or trait-based screening. Large-insert libraries, such as fosmid-based and bacterial artificial chromosome-based genomic libraries, maximize the genomic space^14^, but only if those heterologous genes are expressed in the host. Due to its well-developed genetic toolbox, *Escherichia coli* is the preferred host for screening large-insert genomic libraries. However, expression of heterologous DNA in *E. coli* is limited^15^,^16^ and depends mostly on the recognition of the foreign promoter by the sigma factor subunits of the RNA-polymerase (RNAP) of *E. coli*^1^,^16^,^17^. Thus, enabling *E. coli* to recognize a larger fraction of heterologous promoters would increase the functional sample space and enable efficient screening of heterologous DNA libraries, a strategy identified as an important goal for enabling efficient screening of metagenomic libraries^1^. While a known and long standing issue, no reports have described success to this end, to the best of our knowledge.

Here we report a strategy to enable effective screening of heterologous genomic libraries in *E. coli* by expressing heterologous sigma factors. Our hypothesis is that, when expressing heterologous sigma factors, the core RNAP of the host (here *E. coli*) can be recruited to initiate the transcription from heterologous promoters. We show that the expression of the *Lactobacillus plantarum* (*Lpl*) RpoD increases the functional genomic space in *E. coli*, as quantified by green fluorescence protein (GFP) expression using five heterologous genome-wide, promoter GFP-trap libraries. *Lpl* RpoD increases the GFP^+^ population in all the five libraries tested, which were constructed from phylogenetically diverse genomes, namely, those of *Lpl*, *Bacillus subtilis* (*Bsu*), *Deinococcus radiodurans* (*Dra*), *Clostridium pasteurianum* (*Cpa*) and *C. acetobutylicum* (*Cac*). Furthermore, we demonstrate that the concept works well for a metagenomic library composed of DNA extracted from soil. As a proof of concept for how such effective screening of heterologous genomic libraries can be applied to generate novel complex traits, we expressed a fosmid-based *Lpl* genomic library with large inserts and then screened for genetic loci imparting ethanol tolerance to *E. coli*. We show increased transcripts from heterologous genes located on a fosmid imparting ethanol tolerance. Ethanol tolerance is a complex trait of industrial importance^18^ that has attracted considerable attention^19^,^20^,^21^,^22^, and has been also employed as a model phenotype for the tool development^11^,^19^,^20^,^21^,^22^,^23^,^24^,^25^. *Lpl* is one of the most ethanol, butanol and generally alcohol and solvent-tolerant organisms known^26^,^27^,^28^,^29^. Our strategy can increase the efficiency of genomic library screening to facilitate the discovery of novel genetic elements from otherwise inaccessible genomes.

## Results

### GFP-trap libraries assess recognition of heterologous promoters

**Figure 1:**
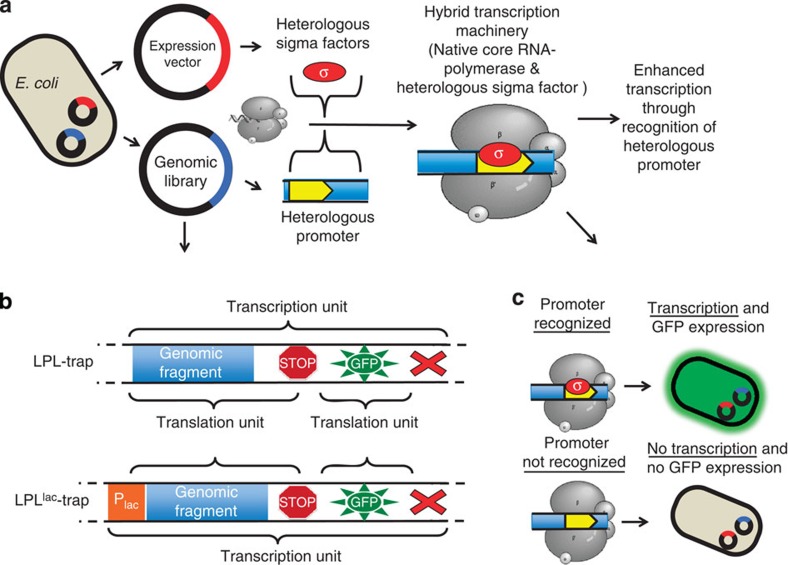
Concept and Strategy. (**a**) A heterologous transcription factor expressed in the cell recruits the native core RNA-polymerase and initiates transcription from heterologous promoters present on genomic library inserts leading to increased transcription. (**b**) Two promoter GFP-trap libraries, LPL-trap and LPL^lac^-trap libraries, were constructed to assay transcription from heterologous promoters. A small *Lpl* genomic fragment of about 726 bp was fused to a *gfp* reporter gene as a single transcriptional unit. (**c**) If transcription inside the genomic fragment occurs, the cell expresses GFP, which can be measured in a high throughput fashion via flow cytometry. Transcription and, therefore, GFP expression is initiated by P_lac_ located in front of the genomic insert for the LPL^lac^-trap library (**b**).

We desired to assess, in a quantitative and high-throughput way, the fraction of heterologous promoters that can be recognized by the *E. coli* RNAP to initiate transcription. To this effect, for each of five phylogenetically diverse genomes, we constructed promoter GFP-trap libraries (Fig. 1b), similar to what was previously described^30^. The five genome-wide heterologous libraries were LPL-trap, BSU-trap, DRA-trap, CPA-trap and CAC-trap libraries, which were constructed from the *Lpl*, *Bsu*, *Dra*, *Cpa* and *Cac* genomes, respectively (Table 1). For clarity, we describe the construction and properties of these libraries based on the LPL-trap and LPL^lac^-trap libraries. The latter was constructed from the *Lpl* genome as a positive control to quantify transcriptional termination within the genomic fragments, and serves as a validation for the proposed concept (described below and in Supplementary Note 1).

**Figure 2:**
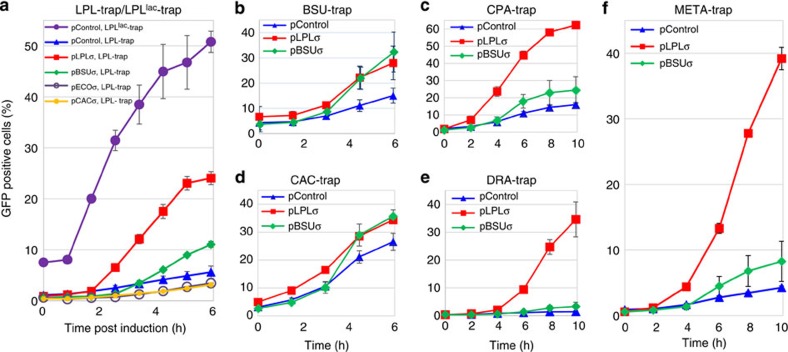
GFP expression profiles of promoter GFP-trap libraries. (**a**) GFP profiles of the LPL^lac^-trap library (in NEB10beta(pControl), purple circles) and LPL-trap library in the plasmid control strain NEB10beta(pControl) (blue triangles), the *Lpl rpoD* expression strain NEB10beta(pLPLσ) (red squares), the *B. subtilis sigA* expression strain NEB10beta(pBSUσ) (green diamonds), the *C. acetobutylicum sigA* expression strain NEB10beta(pCACσ) (orange circle) and the *E. coli rpoD* expression strain NEB10beta(pECOσ) (grey open circles). (**b–e**) GFP profiles of the BSU-trap (**b**), CPA-trap (**c**), CAC-trap (**d**), DRA-trap (**e**) and META-trap (**f**) libraries in the plasmid control strain NEB10beta(pControl) (blue triangles), the *Lpl rpoD* expression strain NEB10beta(pLPLσ) (red squares) and the *B. subtilis sigA* expression strain NEB10beta(pBSUσ) (green diamonds). Error bars represent the s.d. of ≥3 biological replicates.

LPL libraries were constructed from randomly sheared fragments of genomic *Lpl* DNA with an eightfold genomic coverage (Methods). Sequencing of 10 randomly selected inserts confirmed an average insert size of 726 bp (Table 1), purposefully chosen to be smaller than the average gene size in prokaryotes (of about 924 bp (ref. 31)) to maximize the number of DNA fragments that contain promoters that are not followed by transcriptional terminators (Supplementary Note 1). The library insert was fused in front of a promoterless GFP gene (*gfp*; Fig. 1b), which was optimized for translation by incorporating three frame stop codons and a ribosomal binding site in front of the gene. Thus, transcription initiated inside a library insert leads to expression of *gfp* and the resulting green fluorescence is used as a direct measure of transcription from *Lpl* promoters. Flow cytometry (FC) analyzes this fluorescent signal from individual library clones (Fig. 1c) and, thus, the expression profile of the libraries can be acquired in a high-throughput fashion to quantify the fraction of *Lpl* promoters recognized by *E. coli.* Random fragmentation of genomic DNA (gDNA) generates a collection of different inserts containing promoters, terminators as well as DNA of open-reading frames (Supplementary Fig. 1). We first tested the validity of our FC assay by analysing the GFP expression profile of the LPL^lac^-trap library (Fig. 2a). Here the isopropylthiogalactoside (IPTG)-inducible *E. coli lac* promoter, P_lac_, is placed upstream of the library insert to initiate transcription leading to GFP expression if no terminator is present in the insert. We performed a simulation based on the LPL-trap and LPL^lac^-trap libraries (Supplementary Note 1) and we estimated that 62% of the LPL^lac^-trap fragments would lead to GFP expression on IPTG induction. Experimentally, we observed that the fraction of GFP-expressing cells increased steadily to a maximum of 54%, 7 h post induction (Fig. 2a). While lower than predicted (see discussion in Supplementary Note 1), this demonstrates that our FC assay is conservatively valid.

To establish the baseline of *Lpl* promoter expression in unmodified *E. coli*, we determined the fraction of library inserts containing an *Lpl* promoter that is recognized by the native *E. coli* RNAP following the GFP expression profile of the LPL-trap library when co-transformed with the control plasmid (pControl). A maximum of 6.5% of the library population became GFP-positive 7 h post induction (Fig. 2a), indicating that some *Lpl* promoters are recognized by the native *E. coli* RNAP.

### Heterologous sigma factors enable recognition of foreign promoters

Using the LPL-trap library, we investigated whether expressing the major sigma factor of *L. plantarum* (RpoD) can increase the fraction of *Lpl* promoters recognized by *E. coli* (Fig. 1a). *Lpl rpoD* is one of only three sigma factor genes in the *Lpl* genome and whose regulon encompasses 99% of all *Lpl* genes The LPL-trap library was co-transformed into *E. coli* together with the *Lpl rpoD* expressing plasmid (pLPLσ) and the GFP profile of the resulting library population was followed after induction of *Lpl rpoD* expression. *Lpl rpoD* expression was verified via PCR with reverse transcription (RT–PCR; Supplementary Note 2; Supplementary Fig. 2). A steady increase of GFP-positive cells was observed (Fig. 2a) reaching, 7 h post induction, a maximum of 23%, which represents a 3.5-fold increase of GFP-expressing cells compared with the plasmid control strain (pControl; Fig. 2a). Simulation analysis (Supplementary Note 1) estimates that 25% of all inserts in LPL-trap library carry an *Lpl* promoter upstream of *gfp*, which, if recognized by the host, would result in GFP expression. Thus, this 23% fraction compares favourably with the predicted maximum of 25% (see above and Supplementary Note 1) and suggests that most of the *Lpl* promoters in the library can be recognized by the engineered *E. coli* strain. To rule out the possibility that the *Lpl* RpoD initiates transcription from the backbone of the GFP-trap library vector, we tested GFP-trap plasmids that contained random synthetic DNA fragments as inserts, which contained neither a promoter nor a terminator and the GFP^+^ population was small (Supplementary Note 3; Supplementary Fig. 3).

To investigate whether this increased promoter recognition is the specific result of expressing *Lpl rpoD* or whether overexpression of any sigma factor could have the same effect, expression plasmids containing the major sigma factors from *E. coli* (pECOσ, expressing the *E. coli rpoD*), *Cac* (pCACσ, expressing the *Cac sigA*) and *Bsu* (pBSUσ, expressing the *Bsu sigA*) were individually transformed into *E. coli* together with the LPL-trap library and the GFP profiles examined (Fig. 2a). Overexpression of either *E. coli* RpoD or *Cac* SigA resulted in decreased *Lpl* promoter recognition. However, expression of *Bsu* SigA together with the LPL-trap library led to increased GFP expression up to 11% (Fig. 2a). While not as substantial an increase as with pLPLσ, this suggests that cross recognition of heterologous promoters by different sigma factors is possible; this is pursued further below. We also demonstrated that chromosomal integration of *Lpl rpoD* (Supplementary Note 4; Supplementary Figs 4 and 5) leads to enhanced *Lpl* promoter recognition, although fractionally less than what was achieved by plasmid-based *Lpl rpoD* expression. Furthermore, we found that expression of an alternative *Lpl* sigma factor (RpoN, with a 21 gene regulon^32^) slightly but not significantly (*t*-test *P*-value=0.089) further increased *Lpl* promoter recognition in *E. coli* (Supplementary Note 5; Supplementary Fig. 4).

### Cross species promoter recognition

Metagenomic libraries generated from a mixed population of organisms contain a large genomic diversity of promoters. Thus, effective screening of such libraries requires the recognition of a large set of such diverse promoters. We examined whether *E. coli* transcription from heterologous promoters from multiple organisms (*Lpl*, *Bsu*, *Cpa*, *Dra* and *Cac*) could be initiated by expressing one or a few foreign sigma factors; this would require substantial promoter cross recognition. To do so, we co-transformed a sigma factor expression plasmid (pLPLσ or pBSUσ) along with an individual library (BSU-trap, CPA-trap, CAC-trap and DRA-trap) and monitored GFP expression (Fig. 2b–e). We observed a large range in the baseline of the GFP^+^ population with the control (empty) pControl strain (Fig. 2b–e), likely due to the phylogenetic distances between these organisms (including their G+C content) and the average library insert size (Table 1, discussion in Supplementary Note 6). However, in each case, the strain expressing *Lpl rpoD* showed a large increase in promoter recognition compared to control. Likewise, the strain expressing *Bsu sigA* showed a statistically significant improvement over the control in all libraries. The *Lpl rpoD* expressing strain outperformed the *Bsu sigA* except for equivalent performance on BSU trap.

In addition, we also tested the impact of expressing the main sigma factors from *Deinococcus radiodurans*, *Lactococcus lactis* subsp. *lactis* and *Lactobacillus brevis* (the latter two containing only three sigma factors each, like *Lpl*). In each of these cases, however, no increased GFP^+^ population was observed for any of the GFP-trap libraries (Supplementary Fig. 6) showing the unique capabilities of *Lpl* RpoD.

Overexpressing multiple heterologous sigma factors did not provide a benefit in terms of expanding promoter recognition (Supplementary Note 7), and significantly, had a profound negative effect on *E. coli* growth (Supplementary Note 8; Supplementary Fig. 7).

To generalize the concept and its impact, using DNA extracted from soil, we constructed a metagenomic GFP-trap library, which contained prokaryotic and fungal DNA and had a high (61%) G+C content (Methods). The *Lpl rpoD* expressing strain showed the highest GFP^+^ population, ninefold higher than the control; the *Bsu sigA* expressing strain was nearly double the control (Fig. 2f). This powerfully demonstrates that the *Lpl* RpoD sigma factor can be used to access a wide range of promoters originating from different species.

### Expression of *Lpl rpoD* alters *E. coli's* transcriptional program

**Figure 3:**
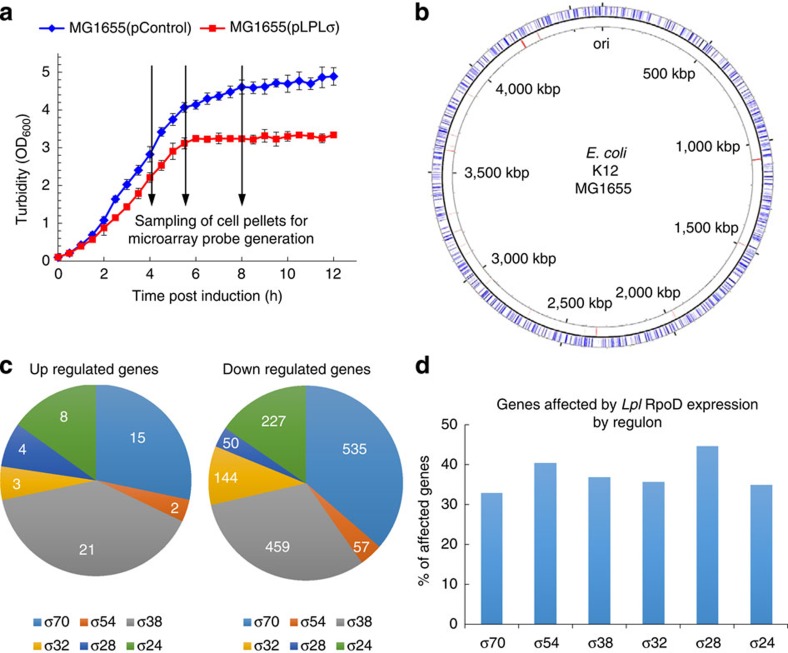
Transcriptional analysis to assess the impact of *Lpl rpoD* expression on the native *E. coli* transcriptional program. (**a**) Growth profile of strain MG1655(pLPLσ) expressing *Lpl rpoD* (red squares) and the control strain MG1655(pControl) (blue diamonds). Microarray sample points are indicated by arrows. Error bars represent the s.d. of two biological replicates. (**b**) Chromosomal map of *E. coli* MG1655 with positions of significant altered genes (blue outer ring: downregulated; red inner ring: upregulated). (**c**) Classification of altered genes based on *E. coli* sigma factor regulons. (**d**) Relative amount of altered genes in each *E. coli* sigma factor regulon.

Our data (Fig. 2) show that *Lpl* RpoD recruits the *E. coli* RNAP components to initiate transcription from *Lpl* and other heterologous promoters. We observed a negative effect on growth in the *Lpl rpoD* expression strain, similar to what was observed with overexpression of the native RpoD (Supplementary Note 8). This suggests that *Lpl* RpoD competes with native *E. coli* sigma factors to recruit the RNAP complex. One would expect that this competition would affect the native *E. coli* transcriptome. We investigated whether this would affect primarily the regulon of the *Lpl* RpoD counterpart, *E. coli* sigma factor 70, or all sigma factor regulons. Microarray-based transcriptional analysis was carried out to compare the transcriptional programs of *E. coli* MG1655 strains carrying pLPLσ and pControl, respectively. The two strains were grown in parallel under IPTG induction, and samples were taken in exponential, transitional and stationary culture phases (Fig. 3a) for microarray analysis, whereby significant expression differences were identified via a one-class time course SAM analysis (Methods). With a ∼5% false-discovery rate, 1710 genes, corresponding to about 40% of all genes in *E. coli* MG1655, had significantly altered expression. The majority (as expected) of >1,600 genes was downregulated and only 50 genes were upregulated. These genes were distributed over the whole genome (Fig. 3b) and belonged to all known sigma factor regulons of *E. coli* (Fig. 3c). The fraction of genes from each sigma factor regulon affected by *Lpl rpoD* expression was between 30 and 45% (Fig. 3d). These data show that expression of *Lpl rpoD* introduces a large perturbation of the native *E. coli* transcriptome, but that the impact is uniformly distributed across all *E. coli* sigma factor regulons.

### Identifying heterologous genetic loci imparting ethanol tolerance

**Figure 4:**
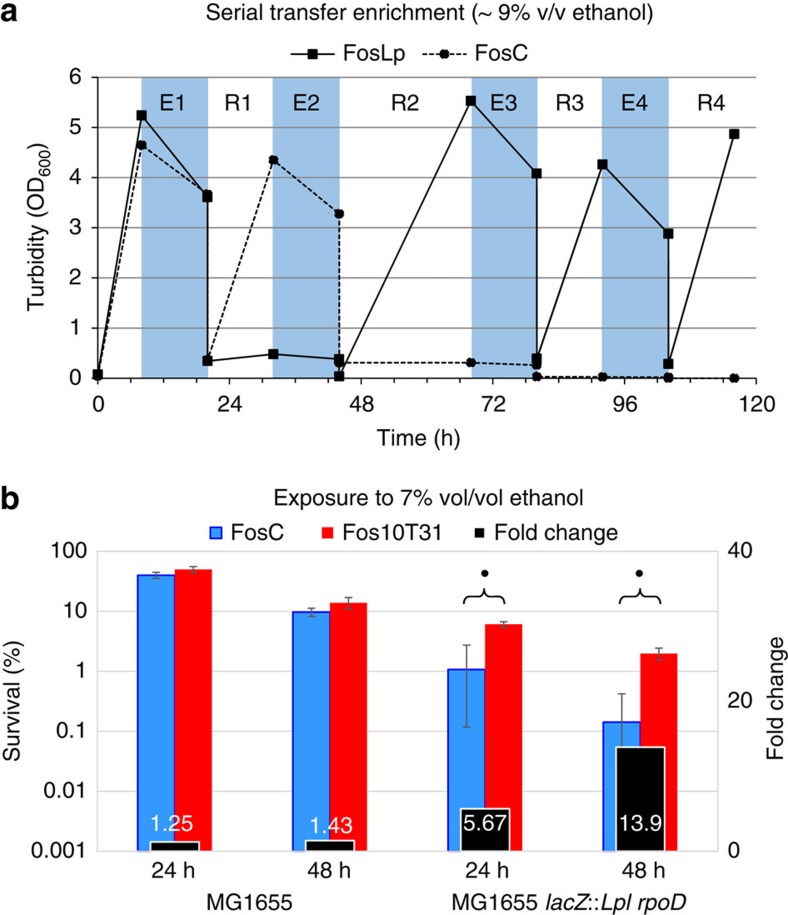
Isolation of ethanol-tolerant clone from fosmid library selection. (**a**) Selection of ethanol-tolerant *E. coli* strains by applying a serial transfer-enrichment strategy consisting of ethanol exposure phases (light blue time periods) and recovery phases (white time periods). The enrichment protocol was performed in parallel for the two cultures: the fosmid-library culture (MG1655(pLPLσ-2, FosLp), squares and solid lines) and the control culture (MG1655(pLPLσ-2, FosC), triangles and dashed lines). A clone (MG1655(pLPLσ-2, Fos10T31)) with increased ethanol tolerance was isolated after plating a serial dilution following the third exposure phase. (**b**) Characterization of the ethanol-tolerant *E. coli* strain carrying Fos10T31. The survival rates of the strain containing the control fosmid, FosC (blue bars) and Fos10T31 (red bars) were determined after 24 and 48 h of exposure in both MG1655 and the *Lpl rpoD* expression strain (MG1655 *lacZ*::*rpoD*). The fold change difference in survival is shown (black bars). Error bars represent the s.d. of three biological replicates. Dot indicates two-tailed *t*-test *P*-value<0.05.

We desired to demonstrate that increased transcription of genes from the ethanol tolerant *L. plantarum* facilitated by the expression of *Lpl* RpoD could be employed to select *Lpl* genetic loci enabling a selectable trait. To this effect, we expressed in wild-type (WT) *E. coli* MG1655 the *Lpl* fosmid library FosLp with an insert size of about 35 kbp, as well as the control fosmid FosC in tandem with *Lpl rpoD* from plasmid pLPLσ-2 (Table 1; pLPLσ-2 differs from pLPLσ in origin of replication due to compatibility issues with the fosmid library). Cultures of the library MG1655(FosLP/pLPLσ-2), as well as of the control strain MG1655(FosC/pLPLσ-2), were screened in parallel via a serial-enrichment strategy consisting of alternating rounds of ∼9% (v/v)-ethanol exposure and recovery phases of 12–24 h (Fig. 4a). Following an initial cultivation for 7 h with IPTG induction to express *Lpl rpoD* and, thus, most of the *Lpl* genes, ethanol was added to ∼9% v/v concentration. After 12 h of exposure to ethanol, 10 ml of cultures were transferred into fresh media and allowed to recover for 12–24 h until the next ethanol exposure. Serial dilutions were plated after each phase to isolate individual clones and growth as well as ethanol concentrations were monitored throughout the serial enrichment. Two culture replicates of the control strain MG1655(FosC/pLPLσ-2) died out after the second or third exposure. Clones with increased survival were isolated after the third exposure phase from the MG1655(FosLp/pLPLσ-2) culture. Among 18 clones tested (15 of which showed increased tolerance, 2 sequenced and found unique) for survival in 7% v/v ethanol (Methods), clone 10T31 (Fos10T31) consistently showed the highest survival. Sequencing Fos10T31 revealed a 30-kb insert spanning the *Lpl* chromosomal locus 3,176,475-3,206,327 (NCBI Ref-Seq accession code NC_004567) containing several annotated genes with a potential role in ethanol tolerance. Ethanol tolerance is a complex phenotype involving several potential mechanisms, including mechanisms involving stress proteins, molecular pumps, DNA-repair proteins, altered membrane properties and energy metabolism^18^,^20^,^23^. Here we found genes encoding transporters (*araP*, lp_3563 and lp_3565), two membrane proteins (lp_3575 and lp_3577), proteins associated with energy metabolism (*lox* and *pox4*), as well as two proteins (catalase (*kat*)) and a heat-shock protein (*clpL*)) involved in stress response.

To verify that the observed ethanol tolerance is not the result of spontaneous mutations introduced during the ethanol exposure, Fos10T31 and FosC were retransformed into *E. coli* MG1655 *lacZ::rpoD* (*rpoD* integration was to ensure stable strains for tolerance assays) never exposed to ethanol. Ethanol survival of *E. coli* MG1655 *lacZ::rpoD* expressing Fos10T31 was 5.7-fold and 13.9-fold higher after a 24 and 48 h exposure to 7% (v/v) ethanol, respectively, than the control strain expressing FosC (Fig. 4b). Thus, the observed ethanol tolerance is a specific effect of the genes on Fos10T31. Furthermore, the effect of Fos10T31 was tested in the WT MG1655. Without *Lpl rpoD* expression, the increase in tolerance of the Fos10T31 strain compared with the FosC strain to 7% ethanol was reduced to 1.3-fold and 1.4-fold (statistically insignificant, *t*-test *P*-value>0.05, ) at 24 and 48 h, respectively. Thus, *Lpl rpoD* expression greatly increases the screening sensitivity to identify beneficial genomic loci. Note that the absolute survival in MG1655 *lacZ::rpoD* for both Fos10T31 and FosC is lower than in the WT strain. This reflects the impact of *Lpl rpoD* expression in competing with the *E. coli* sigma factors, and notably with σ^38^ (RpoS), which protects the cells from many toxic-chemical stresses^18^. We found that overexpression of *Lpl rpoD* has a significant effect on growth (Supplementary Note 8), which we hypothesized derives from the competition for the remaining RNAP components between the native and *Lpl* RpoD. To test this hypothesis, we examined the impact of *Lpl* RpoD expression on the expressed regulons of the native *E. coli* sigma factors. Indeed, *Lpl* RpoD expression affected the expression of over a third of the *E. coli* genome, spread evenly over all sigma factor regulons (Fig. 3b–d). This is not, however, a problem in developing the tolerant phenotype in that once the specific *Lpl* genes that impart tolerance are identified with further subcloning, they can be expressed off native *E. coli* promoters without the need for *Lpl rpoD* expression in final strain.

### RNA-seq shows expression of *Lpl* genes is enabled by *Lpl* RpoD

The data of Figs 2 and 3 show that *Lpl* RpoD enables superior expression of genes from *Lpl* libraries, and that increased expression of *Lpl* genes from Fos10T31 imparts ethanol tolerance. To confirm and detail these conclusions, we performed strand-specific RNA-seq transcriptome analysis of *Lpl* genes in *E. coli*. Four cultures were grown in LB in biological duplicates: MG1655(FosC), MG1655(Fos10T31), MG1655 *lacZ::rpoD*(FosC) and MG1655 *lacZ::rpoD*(Fos10T31). These cultures were induced for 6 h with IPTG at which time samples were taken for RNA-seq analysis. RNA was used to make strand-specific RNA-seq libraries, which were sequenced using HiSeq 2500 (Illumina). Reads were aligned to both the *E. coli* MG1655 and *Lpl* WCFS1 genomes and normalized reads per kilobase per million (RPKM) calculated. A negligible number of reads aligned to the *Lpl* genome from samples with FosC (with the exception of MG1655 *lacZ::rpoD*(FosC), where reads aligned to *Lpl rpoD*, as expected).

**Figure 5:**
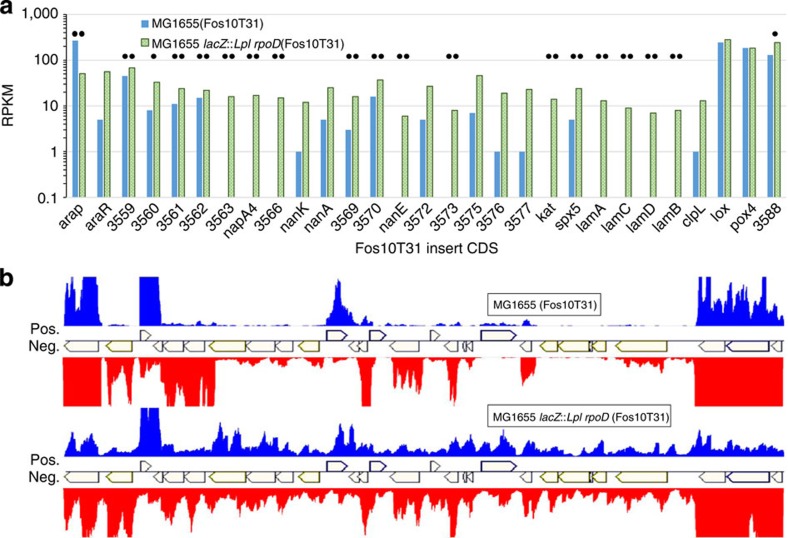
Strand-specific RNA-seq analysis to assess expression of *L. plantarum* genes in *E. coli*. (**a**) Reads per kilobase per million (RPKM mapped sequence reads) of transcripts on Fos10T31 in the control strain MG1655 (blue bars) and MG1655 *lacZ::rpoD* (green bars). Genes listed in order of position on Fos10T31. Dot indicates differential expression (*q*-value<0.05), double dot indicates *q*-value<0.01. (**b**) Representative raw read alignment of transcripts from the Fos10T31 insert sequence. Most genes are coded on the negative strand. Scales for all alignments are identical. Red reads are for transcripts from genes on the negative strand of the *Lpl* DNA, and blue is for transcripts from genes on the positive strand. Compared with the control strain, the impact of *Lpl*-RpoD expression in *E. coli* is profound in generating transcripts from both the strands of *Lpl* DNA.

Out of the 29 *Lpl* genes (most of which are coded on the negative strand) on the Fos10T31 insert, 18 were expressed higher (based on coding strand RPKM, *q*-value<0.05) in MG1655 *lacZ::rpoD*(Fos10T31) compared with MG1655(Fos10T31) (Fig. 5a). Only one gene, *Lpl araP*, was expressed higher in MG1655. Compared with MG1655, three times as many Fos10T31 genes in MG1655 *lacZ::rpoD* had above average RPKM (19.6) values; this is consistent with LPL-trap data where 3.5 times as many clones were GFP positive in the strain expressing *Lpl* RpoD (Fig. 2a). *Lpl* RpoD enables transcription from both coding and noncoding strands of the Fos10T31 insert (Fig. 5b). This is not unusual and takes place natively in most prokaryotic genomes as has been recently reported^33^,^34^. These data also will make possible to select a subset of Fos10T31 genes (those with the highest differential expression between the two strains; Fig. 5b) for screening to identify those responsible for the tolerant phenotype.

We also examined the change in the *E. coli* transcriptome when the *Lpl* RpoD was expressed from a single chromosomal copy. Using our strand-specific RNAseq data, we compared the transcription profiles of MG1655(Fos10T31) and MG1655 *lacZ*::*rpoD*(Fos10T31). Similar to above, we observed differential expression of 41% of *E. coli* genes (1767 genes, *t*-test *P*-value *P*<0.05). Of those, 20% (346 genes) had greater than twofold change in either direction (118 upregulated and 228 downregulated). To address whether expression of *Lpl* RpoD results in different sites of transcription of foreign DNA, we asked whether there were a large number of novel and different transcripts observed in the *Lpl* RpoD expression strain compared with the control. To test, we randomly selected multiple 6-kb regions on the *E. coli* genome and documented the transcription profile from the *Lpl* RpoD expression strains compared with control strains (pre-normalized read density output from RNAseq experiments, Supplementary Fig. 8). While the quantitative output of RNAseq reads varies, the two strain types show extremely similar transcripts and transcription patterns with respect to where transcription is initiated and terminated. That is, the relative abundance of the messenger RNAs (mRNAs) was significantly altered between the two strains, but the same mRNA transcripts were present in both populations.

## Discussion

We demonstrated that, for the first time, the transcription machinery of *E. coli* (and in principle of any other host organism) can be engineered to recognize a large set of heterologous promoters, thus leading to increased expression of heterologous genes. This was successfully exploited for a function-based screening of a fosmid library to identify *Lpl* determinants imparting ethanol tolerance in *E. coli*. The ethanol tolerance imparted by the genes on Fos10T31 expressed in MG1655 *lacZ::rpoD* compares very favourably with other reported tolerant strains in the literature (Supplementary Table 1). For example, Goodarzi *et al.*^19^ report a 1.5–1.6-fold increase in tolerance under similar conditions, while our strain shows a 14-fold higher tolerance compared with control. We also show that most of these genes contained on the fosmid are poorly expressed in the WT host, necessitating an expression strain.

By expressing the *Lpl rpoD* in *E. coli*, we achieved increased *Lpl* promoter recognition up to 3.5-fold (which accounts for the recognition of most *Lpl* promoters) and also markedly increased cross recognition of promoters from heterologous (*Dra*, *Bsu*, *Cpa* and *Cac*) and metagenomic libraries (Fig. 2b–g). Expression of *Bsu* SigA had a similar but less marked impact on cross recognition of heterologous promoters (Fig. 2b–f). In the G+C-rich DRA-trap and META-trap libraries, the data show that the *E. coli* transcription machinery does a poor job of initiating transcription (only 1 and 4% were GFP^+^, respectively; Fig. 2e–f), while the strain expressing *Lpl rpoD* is able to initiate transcription from 35 and 39% *Dra* and metagenomic library clones, respectively. These libraries are a good example of the type of libraries that would be poorly screened without the aid of *Lpl* RpoD expression. Our method can be applied to enable the expression of a large subset of genes found in more complex heterologous or metagenomic libraries^15^,^35^. It may even be possible to tailor strains for efficient screening of specific metagenomic libraries by expressing certain sigma factors, depending on the environmental source of the metagenome and the expected or desired phylogenetic composition.

While the consensus sequence between *E. coli* and *L. plantarum* are similar^36^, the stringency of the binding requirement of particular sigma factors can vary between species, even if the consensus sequences are identical^37^. Here this is complicated by *L. plantarum* RpoD expression outside its native conditions, including related activators and other regulatory elements (for example, small RNAs), which has a profound effect on the transcriptome^38^.

Promoter recognition was assessed with the GFP-trap method, based on the substrate induced gene expression (SIGEX) method of Uchiyama *et al.*^30^. Examining transcription initiation via fluorescent proxy enables a rapid, quantitative screening of thousands of potential promoters from hundreds of thousands of unique genetic segments from across the genomes of heterologous organisms and across genetic elements from the metagenome. This method is more robust than assessing the efficacy of such a system with other proxies such as antibiotic resistance markers or blue/white colony counting.

Here we focused on overcoming limitations in heterologous gene expression at the transcription level. Methods have been reported to overcome limitations at the translation level, such as directed evolution of ribosomal protein S1 to increase translation initiation^39^ or the introduction of transfer RNAs to accommodate rare codons^40^,^41^. We envision expression of heterologous sigma factors as a tool for the functional screening of metagenomic and heterologous genomic libraries. Desired genes and operons, once discovered, can then be optimized via codon optimization, synthetic regulatory structure, choice of strain and so on, but to be found, they must be first transcribed.

## Methods

### Strain and growth conditions

Strains, plasmids and oligonucleotides are listed in Supplementary Tables 2, 3 and 4, respectively. Cultures were performed in lysogeny broth (LB, containing 10 g l^−1^ NaCl, 10 g l^−1^ bacto tryptone and 5 g l^−1^ yeast extract) with antibiotics (100 μg ml^−1^ ampicillin, 35 μg ml^−1^ chloramphenicol for plasmids and 12.5 μg ml^−1^ for fosmids, 50 μg ml^−1^ kanamycin) as required at 37 °C with shaking at 220 r.p.m.

### Construction of destination plasmids

To streamline the cloning of multiple sigma factors as well as the construction of promoter GFP-trap libraries from a variety of organismal or metagenomic DNA (mgDNA), a two-plasmid expression system was constructed. Two compatible plasmids, a low-copy plasmid for sigma factor expression and a high copy plasmid for the promoter GFP-trap libraries, were constructed as destination vectors to utilize the *in vitro* recombination Gateway technique (Invitrogen, Carlsbad, CA, USA). Thus, any desired combination of sigma factor and genomic library can be cloned in the two plasmid expression system without cumbersome genetic manipulation. A detailed description of the construction of the destination plasmids is found in other sections below. (‘Construction of the sigma factor expression plasmids' and ‘Construction of the promoter GFP-trap destination vectors'.) Briefly, *gfp* was first cloned into the multiple cloning site (MCS) of pUC19 (New England Biolabs (NEB), Ipswich, MA, USA) before an LR recombination cassette (Invitrogen) was introduced upstream of *gfp*. The resulting plasmid was designated as pUC-LR-GFP. Destination plasmid pLR-GFP was constructed by removing P_lac_ from pUC-LR-GFP. These two high copy plasmids were used to construct the promoter GFP-trap libraries (see below). Cloning an LR recombination cassette in pACYC-Duet (Novagen Merck KGaA, Darmstadt, Germany) generated the low-copy destination plasmid pACYC-LR for sigma factor expression (see below).

### Construction of sigma factor expression plasmids

Sigma factor-coding genes were first amplified from gDNA of the respective organism. The sigma factor expression set comprises the following plasmids: pLPLσ (expressing *Lpl rpoD* (lp_1962)); pLPL_rpoN (expressing *Lpl rpoN* (lp_0787)); pECOσ (expressing *E. coli rpoD* (b_3067)); pBSUσ (expressing *B. subtilis sigA* (BSU25200)); pCACσ (expressing *C. acetobutylicum sigA* (CA_C1300)); pDRAσ (expressing *Deinococcus radiodurans* RpoD (DR_0916)); pLLALσ (expressing *Lactococcus lactis* subsp. *lactis* RpoD (L0139)); and pLBRσ (expressing *Lactococcus brevis* RpoD (LVIS_0756)). The set generated by recombination with pDEST14 comprises: pLPLσ-2 (expressing *Lpl rpoD*), pBSUσ-2 (expressing *B. subtilis sigA*) and pCACσ-2 (expressing *C. acetobutylicum sigA*). The pENTR-gus vector, which contains a promoterless *gus* gene from *Arabidopsis thaliana*, was recombined with pACYC-LR and pDEST14 to create the control vectors, pControl and pControl2, respectively. Expression of sigma factors was verified via RT–PCR (Supplementary Note 2).

The destination vector for sigma factor overexpression was based on the low copy plasmid pACYC-Duet (Novagen) that was converted to a destination plasmid by introducing an LR-recombination cassette amplified from pDEST40 (Invitrogen) with the primer pair DEST-for and DEST-rev. A portion of the pACYC-Duet backbone was PCR amplified including the p15A origin of replication, the chloramphenicol resistance gene as well as the *lacI* repressor using the primer pair DUET-for and DUET-rev. After phosphorylation of the pDEST40 cassette, a ligation with the partial backbone was transformed into *ccd*B survival cells (Invitrogen). The resulting destination plasmid, pACYC-LR, was tested for functionality by *in vitro* recombination with pENTR-gus as per the manufacturer's suggestion. The recombined plasmid, designated as pControl, was used as a control plasmid.

Sigma factors from different organisms were PCR amplified from gDNA with restriction site overhangs (Supplementary Table 4) and cloned via restriction enzyme cloning in the MCS of pUC19 under the control of P_lac_. Using this plasmid, the sigma factors together with the P_lac_ were amplified again with the primer pair pUC19-for and pUC19-rev. The amplified sigma factors were TOPO-TA cloned into pCR8/GW/TOPO (Invitrogen), an entry plasmid to be used in *in vitro* recombination with pACYC-LR. Sigma factors were amplified from *L. plantarum* gDNA to construct pLPLσ (expressing *rpoD*) and pLPL_RpoN (expressing *rpoN*), *E. coli* gDNA to construct pECOσ (expressing *rpoD*), *B. subtilis* gDNA to construct pBSUσ (expressing *sigA*), as well as *C. acetobutylicum* gDNA to construct pCACσ (expressing *sigA*) after *in vitro* recombination with pACYC-LR. These expression plasmids are compatible with the promoter GFP-trap libraries.

Another set of sigma factor expression plasmids was generated by *in vitro* recombination of the entry vectors with the aforementioned sigma factors and the commercial destination vector pDEST14 (Invitrogen), creating pLPLσ-2 (expressing *rpoD* of *Lpl*), pBSUσ-2 (expressing *sigA* of *B. subtilis*) as well as pCACσ-2 (expressing *sigA* of *C. acetobutylicum*). These expression vectors contain the colE origin and are compatible with the fosmid library as well as with the p15A origin-based expression vectors (pLPLσ, pBSUσ and pCACσ).

PCR amplifications were performed with Phusion polymerase (NEB) as well as Taq polymerase (NEB) according to the manufacturer's suggestions. *In vitro* recombinations were performed using the Gateway technology (Invitrogen) according to the manufacturer's suggestions. Primers used to amplify the sigma factors and to construct the plasmids are listed in Supplementary Table 4.

### Construction of the promoter GFP-trap destination vectors

Promoter GFP-trap vectors were constructed as destination plasmids to facilitate a streamlined generation of promoter GFP-trap libraries of various species using the Gateway technology (Invitrogen). A *gfp* gene amplified from pLenti7.3/V5-GW/lacZ (Invitrogen) was cloned via restriction cloning using BamHI and EcoRI into the MCS of pUC19 (NEB). The *gfp* was amplified with primers containing the appropriate restriction sites as well as an optimized ribosomal binding site (RBS) and a stop codon upstream of *gfp* as overhangs (GFP-for and GFP-rev). In a second cloning step, an LR-cassette amplified from pDEST14 (Invitrogen) was cloned upstream of *gfp* using HindIII and SphI. The cassette was amplified again with overhang primers containing the restriction sites as well as a three frame stop signal downstream of the cassette (LR-for and LR-rev). The generated GFP-trap vector was named pUC-LR-GFP and was used to generate the LPL^lac^-trap library. A second GFP-trap vector, designated as pLR-GFP, was constructed by removing the P_lac_ via digest with PciI and HindIII followed by autoligation. This plasmid was used to construct the other GFP-trap libraries. Confirmation of the correct assembly of both destination plasmids was verified by restriction digest and sequencing. Primers used to construct these plasmids are listed in Supplementary Table 4.

### Construction of the promoter GFP-trap libraries

gDNA was isolated using the ChargeSwitch gDNA Mini Bacteria Kit (Invitrogen) according to the manufacturer's instructions from a *Lactobacillus plantarum* WCFS1 culture grown to an optical density (OD_600_) of 1. Approximately 25 μg of this gDNA was sheared on ice with a Nebulizer (Invitrogen) for a total of 4 min in 30-s intervals at 15 psi. The sheared DNA was separated on a 1% agarose gel stained with SYBRGold (Applied Biosystems) and a band ranging from about 200 bp to 1,000 bp was cut out and purified via the PureLink gel extraction kit (Invitrogen). The extracted DNA was polished as described^8^. The polished DNA was then cloned via TOPO-TA cloning into the pCR8/GW/TOPO vector (Invitrogen) and transformed into TOP10 cells (Invitrogen) according to the manufacturer's instructions. Individual transformations were pooled after 1 h outgrowth in SOC media and transferred into LB media containing 100 μg ml^−1^ spectinomycin. On the basis of the colony-forming units (c.f.u.) of a serial dilution plated after inoculation, the library was estimated to contain 1.1 × 10^5^ clones. The library culture was incubated until exponential growth phase (OD of about 1) before a part was resuspended in 15% glycerol LB and stored as 1-ml aliquots at −85 °C. From another part of the library, plasmid DNA (pLPL-entry) was extracted via the QIAprep Spin Miniprep Kit (Qiagen). This plasmid DNA was used for an *in vitro* recombination reaction (Invitrogen) as per the manufacturer's suggestions with pUC-LR-GFP and pLR-GFP to generate the LPL^lac^-trap and LPL-trap libraries, respectively. The recombination reaction mix was transformed into NEB 5-alpha F'I^q^ cells (NEB) for the LPL^lac^-trap library and NEB 10 beta cells (NEB) for the LPL-trap library. After an initial outgrowth in SOC media, transformants from each library were pooled and further outgrown as described for the LPL-entry library, whereby ampicillin instead of spectinomycin was used.

Through serial dilutions, both libraries were estimated to contain about 1.2 × 10^6^ individual clones. Ten individual clones were picked from the LPL-trap library and sequencing revealed an average insert size of 726 bp. The generated promoter GFP-trap libraries were used to transform strains containing different sigma-factor expression vectors or the corresponding control vector. The libraries were introduced via electroporation into sigma factor expressing strains, which were then outgrown as described for the LPL-entry library. These electroporation outcomes generated libraries larger than 1 × 10^6^ clones.

Similarly, GFP-trap libraries were constructed for *Bacillus subtilis* (BSU), *Deinococcus radiodurans* (DRA), *Clostridium pasteurianum* (CPA) and *Clostridium acetobutylicum* ATCC824 (CAC) using isolated gDNA. The entry library size for each organism, cloned into pCR8/GW/TOPO vector (Invitrogen) and transformed into TOP10 cells (Invitrogen), was 21,733, 49,140, 21,9350 and 2,37,260 for BSU, DRA, CPA and CAC, respectively. Following recombinations into the pLR-GFP plasmid, the GFP-trap library size for each organism was 6,60,000, 1,20,750, 2,44,200 and 5,31,423 for BSU, DRA, CPA and CAC, respectively. The genomic coverage, defined as the probability that the entire genome is represented, was calculated with the following formula *N*=ln(1−*P*)/ln(1−*f*) (ref. 42), where *P* is the coverage probability (set at 95%), *f* is the fraction of insert size relative to the genome and *N* is the number of individual clones required to represent the genome (Table 1). Clones (*n*) of LPL-trap (10), BSU-trap (9), DRA-trap (11), CPA-trap (8) and CAC-trap (10) were sequenced to get an average insert size of 726, 1,684, 736, 562 and 267 bp, respectively. On the basis of the genome sizes of these organisms, we calculated that we require 13,820, 7,505, 13,350, 26,850 and 46,337 clones for 95% coverage of LPL, BSU, DRA, CPA and CAC. Thus, based on the entry library clone count (the coverage limiting step), we estimate an 8.0-fold, 2.9-fold, 3.7-fold, 8.2-fold and 5.2-fold genome coverage for the LPL, BSU, DRA, CPA and CAC-trap libraries. The number of clones obtained after recombination was higher than the number of entry clones, thus genome coverage in the GFP-trap libraries was maintained. Finally, when the libraries were transformed into strains expressing sigma factors, we ensured that the transformants exceeded the number of clones in the entry library to prevent loss of genomic coverage. mgDNA was obtained from soil surrounding Delaware Biotechnology Institute using the PowerSoil DNA Isolation kit from MoBio. Isolated mgDNA was sheared with a Nebulizer kit (Invitrogen) and 500–1,000-bp fragments were isolated via agarose gel purification. The META-trap library was constructed in the same manner as above. The average insert size was 609 bp and the total library size was 6,350 clones

### Construction of the *L. plantarum* fosmid library

An *Lpl* fosmid library with inserts of about 35 kb as well as a control fosmid, designated FosC, were constructed with the CopyControl Fosmid Library Production Kit (Epicentre, Madison, WI, USA) yielding a library size of 4,679 clones, which corresponds to a 16.5-fold 95% genome coverage (Table 1). Fosmids of the initial library were methylated as described^8^ and transformed into MG1655(pLPLσ-2). Genome coverage was conserved throughout the library transformations.

gDNA of *Lactobacillus plantarum* WCFS1 (isolated as described above) was sheared by 30 passages through a 50-μl microsyringe and used as a substrate for the CopyControl Fosmid Library Production Kit (Epicentre) according to the manufacturer's recommendations. DNA fragments were first end-repaired, and fragments of about 35 kb (selected from an agarose gel) were ligated into the pCC1Fos backbone. The ligated fosmids were then introduced into Epi300-T1^R^ via lambda-mediated transfection, and the resulting clones were plated on the LB plates containing 12.5 μl ml^−1^ chloramphenicol. The resulting colonies were pooled, resuspended in 15% glycerol LB and stored in 1-ml aliquots as frozen stocks at −85 °C. A total of 4,679 clones were obtained for this initial fosmid library. The control fosmid FosC was constructed as per the manufacturer's recommendations using the supplied control DNA, a 42-kb fragment of the human X-chromosome.

Because the fosmid library was constructed in a restriction and methylation negative cloning strain (Epi300-T1^R^), fosmid DNA (extracted from the fosmid library) was electroporated into a restriction negative but methylation positive cloning strain (NEB 5alpha). Transformations were pooled and outgrown as described earlier (see ‘Construction of the promoter GFP-trap destination vectors'). On the basis of the c.f.u. of a serial dilution plated after pooling, the library was estimated to contain 1.2 × 10^5^ clones. Fosmid DNA, extracted from this library, was then used to transform the screening strain *E. coli* MG1655 containing pLPLσ-2. Here a library size of 6 × 10^4^ clones was achieved. The control fosmid FosC was also methylated in NEB 5alpha cells and introduced into *E. coli* MG1655 containing pLPLσ-2 to generate the appropriate control strain.

### Chromosomal integration of *Lpl rpoD*

The lambda red system^43^ was used to integrate *Lpl rpoD* into the *lac* locus of the WT *E. coli* strain MG1655. First, a kanamycin resistance gene (Kan^R^) flanked by two FRT sites was amplified from pKD4 (FRT-Kan-for and FRT-Kan-rev primer pair) and inserted via restriction digest cloning with SacI and EcoRI into the MCS of pUC19 (NEB) yielding plasmid pUC-kan. *Lpl rpoD* was amplified from gDNA of *Lactobacillus plantarum* WCFS1 (Lpl-rpoD-for and Lpl-rpoD-rev primer pair) and inserted upstream of the Kan^R^ in pUC-kan via restriction digest cloning with KpnI and SacI yielding pUC-rpoD-kan. From this plasmid, a knock-in cassette was amplified (pUC19-for and pUC19-rev primer pair) composed of the *Lpl rpoD* and the downstream Kan^R^ flanked by the homologous regions of pUC19 to the *lac* locus of *E. coli*. This cassette was electroporated into *E. coli* MG1655 containing the helper plasmid pKD20, carrying the lambda red system and plated on the LB plates containing kanamycin. Colony PCR (lacI-for and lacZ-rev primer pair) was performed to select clones with successful integration of the cassette. Clones with a confirmed integration were transformed with the helper plasmid pCP20 and cured of the kan^R^ via FRT-mediated excision. The resulting markerless knock-in of *Lpl rpoD* (MG1655 *lacZ::rpoD*) was verified via colony PCR (lacI-for and lacZ-rev primer pair) and sequencing. Expression of *Lpl rpoD* was checked via RT–PCR (Supplementary Note 4). Sequences of the oligonucleotides used can be found in Supplementary Table 4.

### Flow cytometry and fluorescent activated cell sorting (FACS)

GFP-trap libraries (1 μg DNA) were transformed into sigma factor expression strains (hosted by *E. coli* NEB 10beta for plasmid-based sigma factor genes or *E. coli* MG1655 for integration-based genes) and grown to late exponential phase (OD_600_≈0.8–1.0) and stored (at 4 °C overnight or −80 °C if longer). Dilution plating was carried out to ensure the maintained library coverage. Library cultures were started with a 2-ml frozen stock or 4% overnight culture and grown in 500-ml baffled flask containing 100 ml LB at 37 °C with shaking at 220 r.p.m. Expression of cloned sigma factors was induced with 1 mM IPTG at an OD between 0.3 and 0.5. Green fluorescence was measured with a photomultiplier tube (PMT) fitted with a 530/30 band-pass filter on a BD FACSAria Special System with the FACSDiva software package (BD, Franklin Lakes, NJ, USA). Flow samples were prepared and analyzed as described^44^. GFP gates were established with control strains either expressing GFP (pUC-GFP, >97.5% GFP^+^ after 4 h) or not (pUC19, <0.1% GFP^+^) (Supplementary Fig. 3a). Samples were analyzed immediately after collection.

FACS was performed to enhance the sensitivity of the LPL trap by decreasing the amount of clones carrying an *Lpl* promoter natively recognized by *E. coli*. The LPL-trap library was sorted after an outgrowth for 7 h to allow clones carrying such a promoter to express GFP. About 5.5 × 10^6^ GFP-negative events were collected in a two-way sort and recultivated. With a 19% recovery rate (calculated from a serial dilution plated after FACS), a total number of about 1 × 10^6^ clones was achieved for the enriched sublibrary, designated as sLPL trap. Sequencing of 10 individual clones revealed independent inserts distributed over the whole genome verifying a good diversity of the sorted sublibrary.

### Serial enrichment for the selection of ethanol-tolerant clones

The methylated *Lpl* fosmid library (FosLp) as well as the control fosmid FosC was transformed in strain MG1655(pLPLσ-2) expressing *Lpl rpoD*. The heterogeneous fosmid library (MG1655(pLPLσ-2, FosLp)) as well as the homogeneous control culture (MG1655(pLPLσ-2, FosC)) were grown in parallel in 500-ml baffled flask containing 100 ml LB. Following inoculation (2 ml of a 15% glycerol frozen stock), cultures were induced after 1 h of growth with 1 mM IPTG to express *Lpl rpoD*. The induced cultures were incubated for 7 h to allow for maximum expression of *Lpl* genes on the fosmids. Ten ml of 100% ethanol were added to the culture. After exposure for 12 h to high ethanol stress, 10% of the culture was transferred into fresh LB media containing IPTG to constantly induce *Lpl rpoD* expression and incubated for 12–24 h to allow the cells to recover from the ethanol stress. After this recovery phase, 10 ml of 100% ethanol was again directly added to start the next ethanol-exposure phase. This process of alternating ethanol exposure and recovery phases was carried out until a clear difference in growth between the library culture (MG1655(pLPLσ-2, FosLP)) and the control culture (MG1655(pLPLσ-2, FosC)) was observed (Fig. 4a). Serial dilutions were plated after each exposure phase to isolate clones.

### Ethanol tolerance assay for strain characterization

Cultures of individual clones grown overnight were used to inoculate 50-ml falcon tubes containing 10 ml LB with IPTG. These tubes were outgrown with open caps for 7 h to express *Lpl rpoD* (and thus *Lpl* genes) before 700 μl of culture was replaced by 100% ethanol. The cultures were incubated for 24 and 48 h with closed caps to avoid ethanol evaporation. Colony-forming units before and after the stress were determined by plating a serial dilution and the specific survival rate of a strain was calculated as c.f.u.(*t*=24 or 48 h)/c.f.u.(*t*=0)*100%.

### Strand-specific RNA-seq and analysis

MG1655+FosC, MG1655::LPLσ+FosC, MG1655+Fos10T31 and MG1655::LPLσ+Fos10T31 (all in duplicate) were grown in LB broth and induced with 1 mM IPTG for 6 h at which time 10-ml culture was sampled for RNA isolation. RNA isolation samples were centrifuged at 4,000 relative centrifugal force (RCF) at 4 °C for 10 min, decanted and stored at −80 °C. Ten μg per sample of RNA extracted with the RNeasy kit (Qiagen) had DNA removed with the DNA-free kit (Life) and was enriched for mRNA with the MicrobExpress kit (Ambion) thrice, according to the manufacturers' protocols. The ScriptSeq v2 (Illumina) kit was used to construct the RNAseq libraries. The fragment length of the libraries was checked using Bioanalyzer (Agilent, Santa Clara, CA, USA) before sequencing.

Deep sequencing using HiSeq2500 (Illumina) with a 75-bp read length resulted in individual library sequence files. Files were processed to remove barcodes, trim adaptors and obtain read counts. Rockhopper software^45^ was used to align raw read files to the *E. coli* and *L. plantarum* genomes (annotations from National Center for Biotechnology Information) and to do differential expression analysis (at *q*-value<0.05). Integrated Genome Viewer was used for visualizing read alignments^46^.

### Gene expression analysis

pLPLσ and pControl were transformed into WT *E. coli* MG1655 and cultured in parallel as described above. Growth was followed by OD measurements and cell pellets for microarray probe generation were collected throughout the time course and stored at −85 °C. Probe generation, microarray hybridization and analysis were performed as described^44^. Briefly, 25 μg of total RNA were primed with 3 μl of random hexamer primers (5 μg μl^−1^; Roche Molecular Biochemicals, Indianapolis, IN, USA) at 70 °C for 10 min, then reversed transcribed at 50 °C overnight with 200U SuperScript III RTase (Invitrogen) and 360 μM aminoallyl labelling mix (dATP, dCTP, dGTP, dTTP and aa-dUTP) containing a 2:3 aa-dUTP:dTTP ratio. Complementary DNA (cDNA) was subsequently purified using a Microcon column (Millipore, Billerica, MA, USA). The cleaned-up cDNA was then labelled using Cy3 and Cy5 dyes (Amersham CyDye Mono-Reactive Dye, GE Healthcare, Pittsburgh, PA, USA) following the manufacturer's recommendations. Two hundred and fifty ng of each labelled cDNA (cy3 and cy5) were hybridized using the Gene Expression Hybridization Kit (Agilent) according to the manufacturer's instructions. Fifteen K *E. coli* Gene Expression Microarrays (Agilent) were hybridized with probes generated from three time points (Fig. 3a) for each of two biological replicate experiments, whereby technical replicates were performed as dye-swap experiments. Normalization was carried out through R using the Bioconductor package^47^,^48^, and genes with a significantly altered expression were identified via a one-class time course SAM analysis^49^.

## Author contributions

S.M.G. developed the concept with E.T.P., constructed expression strains and genomic libraries and conceived, performed and analyzed experiments. N.R.S. constructed expression strains and the metagenomic library and conceived, performed and analyzed experiments including RNA-seq experiment. S.A.N. constructed expression strains and genomic libraries and assisted in the development of the concept. Y.C. aided in the construction of the GFP-trap model. K.P.V. aided in RNA-seq bioinformatic analysis. E.T.P. developed the concept and jointly wrote the manuscript with S.M.G., N.R.S. and S.A.N.

## Additional information

**Accession codes**: The microarray data have been deposited in the Gene Expression Omnibus database with accession codes GSM1635618 to GSM1635629. The RNA-seq data have been deposited in the NCBI database with accession codes SAMN03331729 to SAMN03331736.

**How to cite this article**: Gaida, S.M. *et al.* Expression of heterologous sigma factors enables functional screening of metagenomic and heterologous genomic libraries. *Nat. Commun.* 7:7045 doi: 10.1038/ncomms8045 (2015).

## Supplementary Material

Supplementary Information Supplementary Figures 1-8, Supplementary Tables 1-4, Supplementary Notes 1-8 and Supplementary References

## Figures and Tables

**Table 1:** List and features of libraries.

**Library**	**Source**	**Gram±**	**GC%**	**Average insert size (bp)**	**Fold library coverage (95%)**
LPL-trap	*Lactobacillus plantarum*	**+**	44.5%	726	8.0
BSU-trap	*Bacillus subtilis*	**+**	43.5%	1,684	2.9
CPA-trap	*Clostridium pasteurianum*	**+**	29.8%	562	8.2
DRA-trap	*Deinococcus radiodurans*	**+**	66.6%	736	3.7
CAC-trap	*Clostridium acetobutylicum*	**+**	30.9%	267	5.1
META-trap	Soil metagenomic DNA	NA	∼61%	609	NA
Fos-LPL	*Lactobacillus plantarum*	**+**	44.5%	∼35,000	16.5

NA, not applicable.
