# Supplementary material for: Expression of heterologous sigma factors enables functional screening of metagenomic and heterologous genomic libraries
Source: Nat Commun. 2015 May 6;6:7045. doi: 10.1038/ncomms8045 (PMC4432631; doi:10.1038/ncomms8045)
Supplement: Supplementary Information — Supplementary Figures 1-8, Supplementary Tables 1-4, Supplementary Notes 1-8 and Supplementary References [file ncomms8045-s1.pdf]

Supplementary Figures

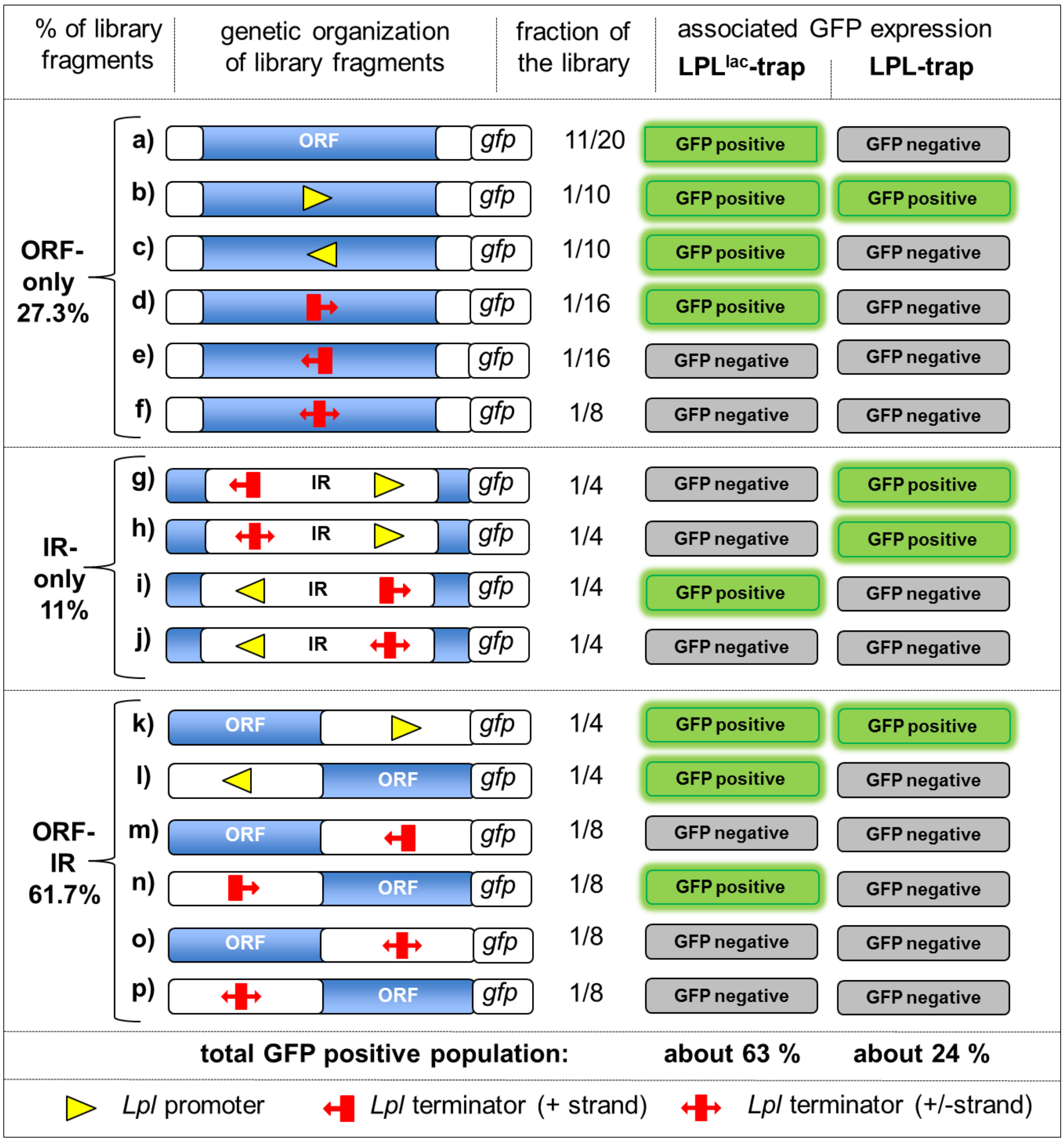

Supplementary Figure 1

Possible genetic makeup of library inserts to estimate the fraction of inserts that may lead to GFP expression in each of the two GFP-trap libraries (LPL^{lac}-trap and LPL-trap).

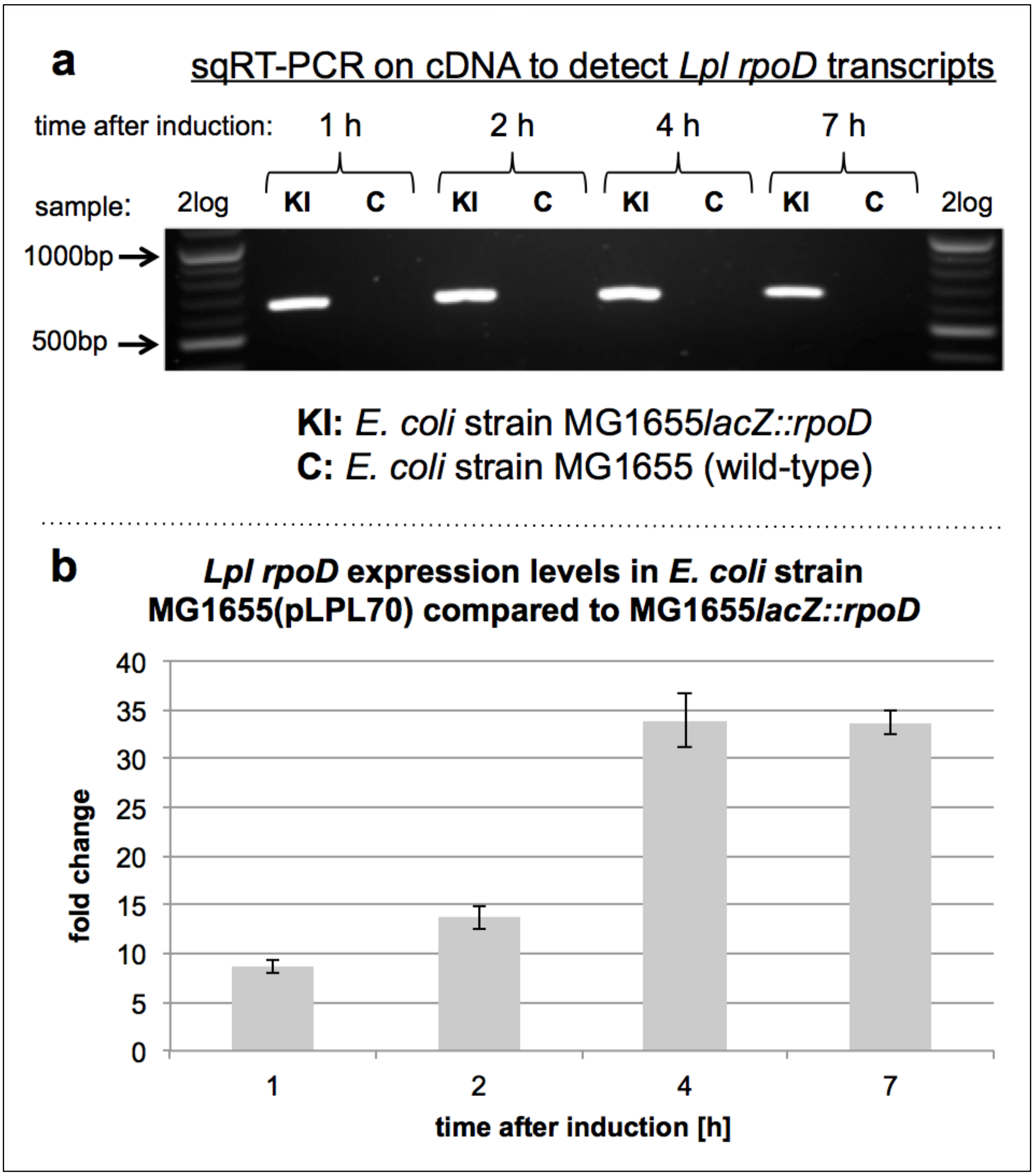

Supplementary Figure 2

Verification of *Lpl rpoD* expression. *Lpl rpoD* mRNA was detected via sqRT-PCR in strain MG1655*lacZ::rpoD* but not in the wild-type strain MG1655, as shown in (**a**). Higher transcription levels (shown as fold change in (**b**)) were detected for the plasmid-based (MG1655(pLPLσ)) versus the chromosomal-based expression of *Lpl rpoD* (MG1655*lacZ::rpoD*). Error bars represent the standard deviation of 3 replicates.

a)

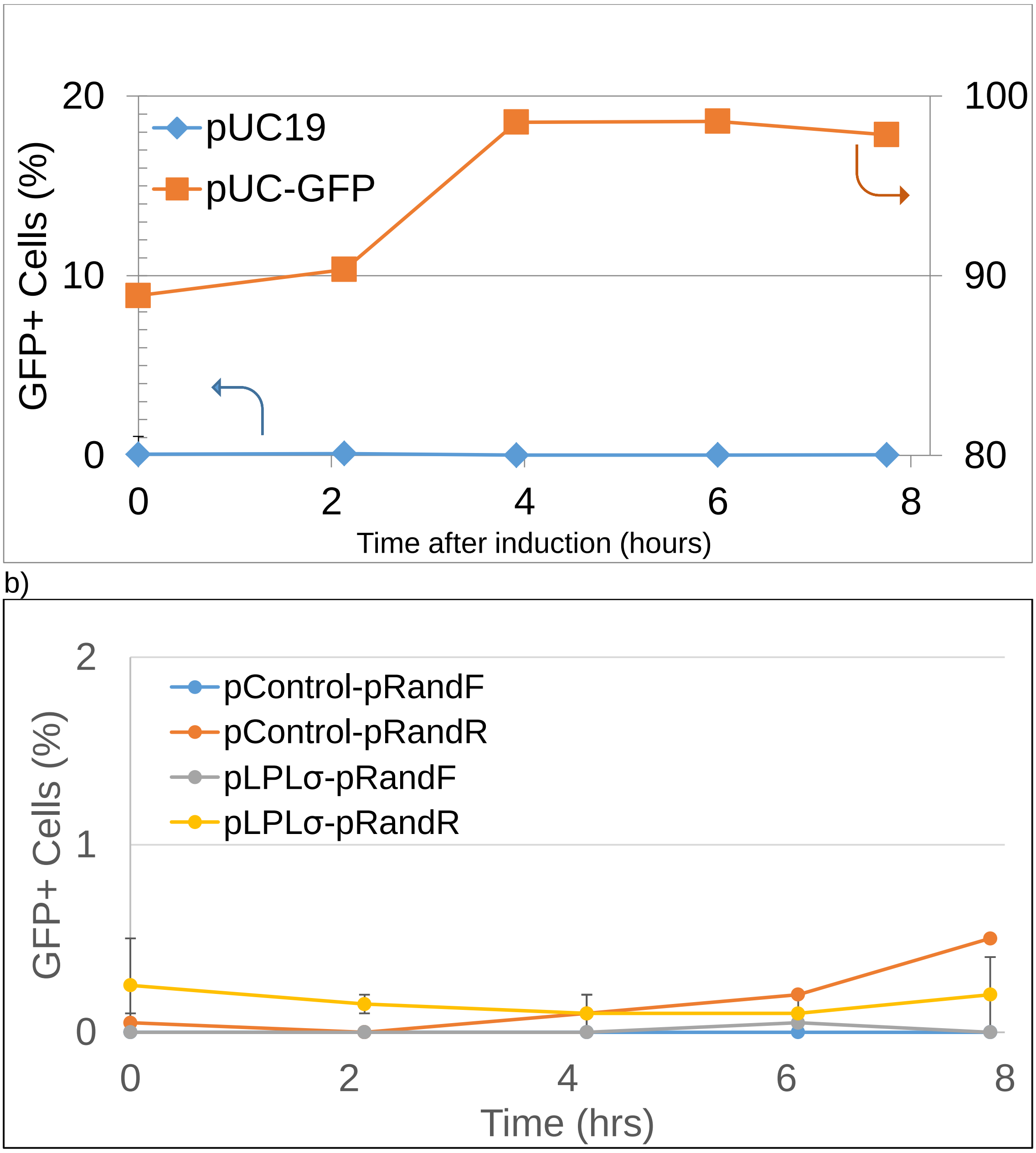

Supplementary Figure 3

GFP profiles of control strains. A) GFP profile of strains containing pUC19 (blue diamonds, primary axis) or pUC-GFP (orange squares, secondary axis) inducted at t=0 with 1 mM IPTG. B) GFP profiles of strains containing the GFP trap plasmids with the random insert in either direction co-transformed with either pControl or pLPLσ. Error bars represent range of biological duplicates.

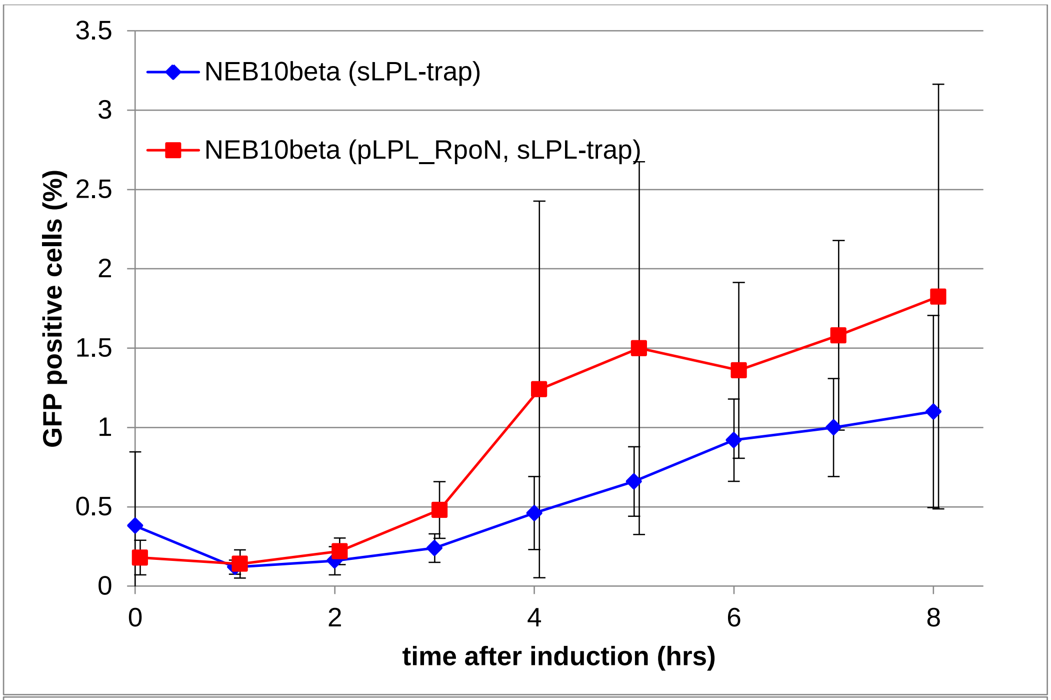

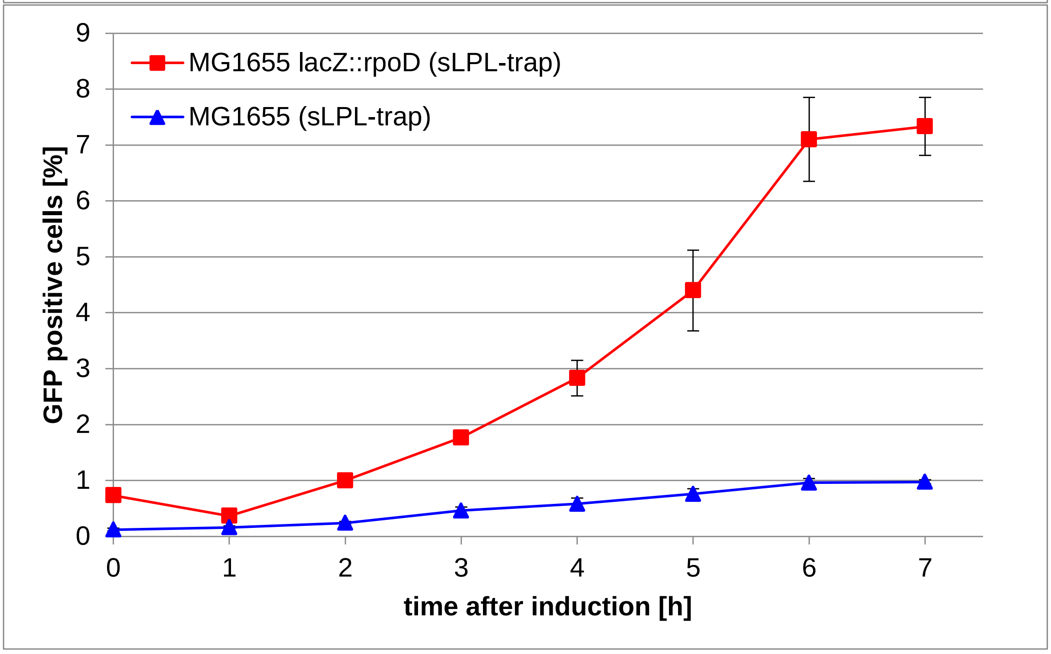

Supplementary Figure 4

GFP expression profiles of the sorted promoter GFP-trap library sLPL-trap with tandem expression of *Lpl rpoD* (MG1655*lacZ::rpoD*) as well as *Lpl rpoN* (NEB10beta(pLPL54). (a) GFP profiles of the strain NEB10beta(sLPL-trap) (blue) and strain NEB10beta(pLPL_RpoN, sLPL-trap) (red); (b) GFP profiles of the strain MG1655(sLPL-trap) (blue) and strain MG1655*lacZ::rpoD*(sLPL-trap) (red). Error bars represent the standard deviation of ≥3 biological replicates.

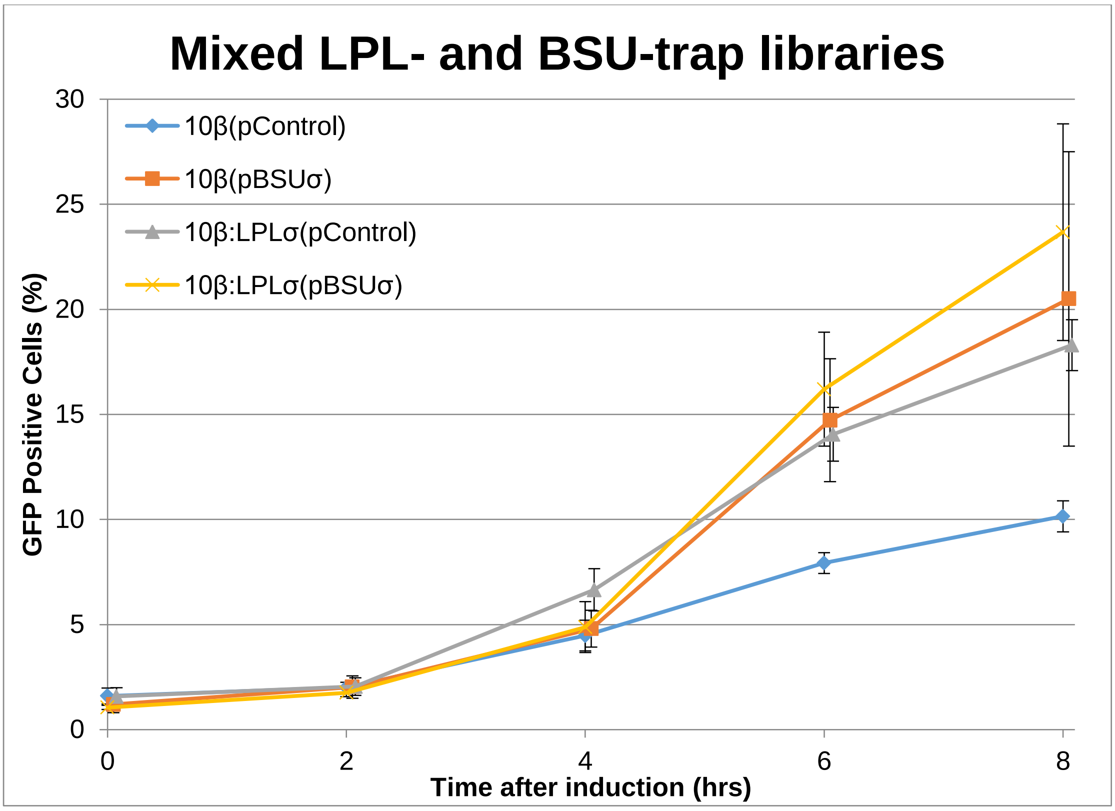

Supplementary Figure 5

GFP profiles of the mixed LPL-trap and BSU-trap library in the plasmid control strain NEB10beta(pControl) (blue diamonds), the *Bsu sigA* expression strain NEB10beta(pBSUσ) (orange squares), the chromosomally integrated Lpl rpoD expression strain NEB10beta:LPLσ(pControl) (grey triangles), and the dual sigma factor expression strain NEB10beta:LPLσ(pBSUσ) (yellow x’s). Error bars represent the standard deviation of ≥3 biological replicates.

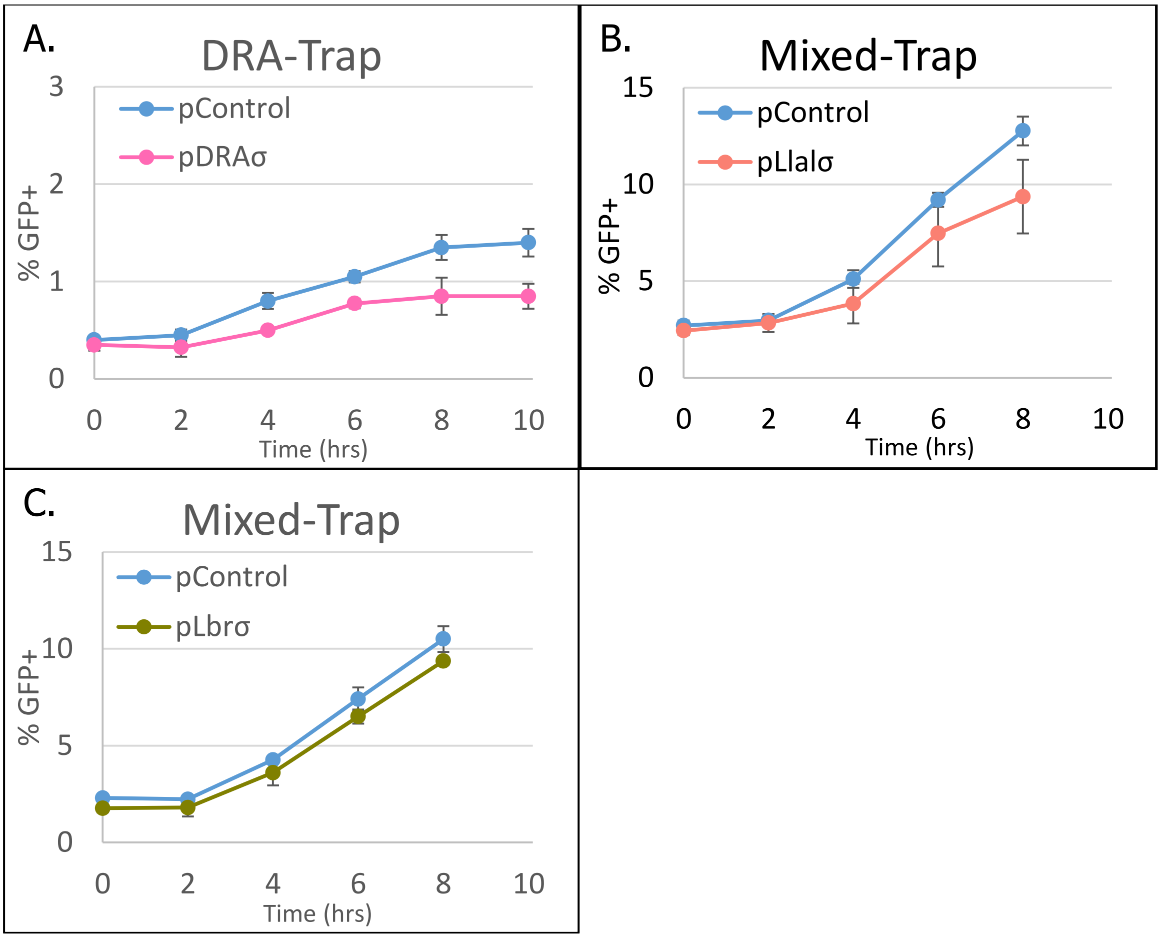

Supplementary Figure 6

GFP expression profiles. (A) GFP profiles of the DRA-trap library in control strain NEB10beta(pControl) (blue) and the *Dra rpoD* expression strain NEB10beta(pDRAσ) (pink). (B) GFP profiles of the Mixed-trap library (equal parts LPL-trap, BSU-trap, CPA-trap, and DRA-trap) in control strain NEB10beta(pControl) (blue) and the *Lactobacillus lactis subsp. lactis rpoD* expression strain NEB10beta(pLlalσ) (salmon). (C) GFP profiles of the Mixed-trap library in control strain NEB10beta(pControl) (blue) and the *Lactobacillus brevis rpoD* expression strain NEB10beta(pLbrσ) (olive). Error bars represent the standard deviation of 3 biological triplicates.

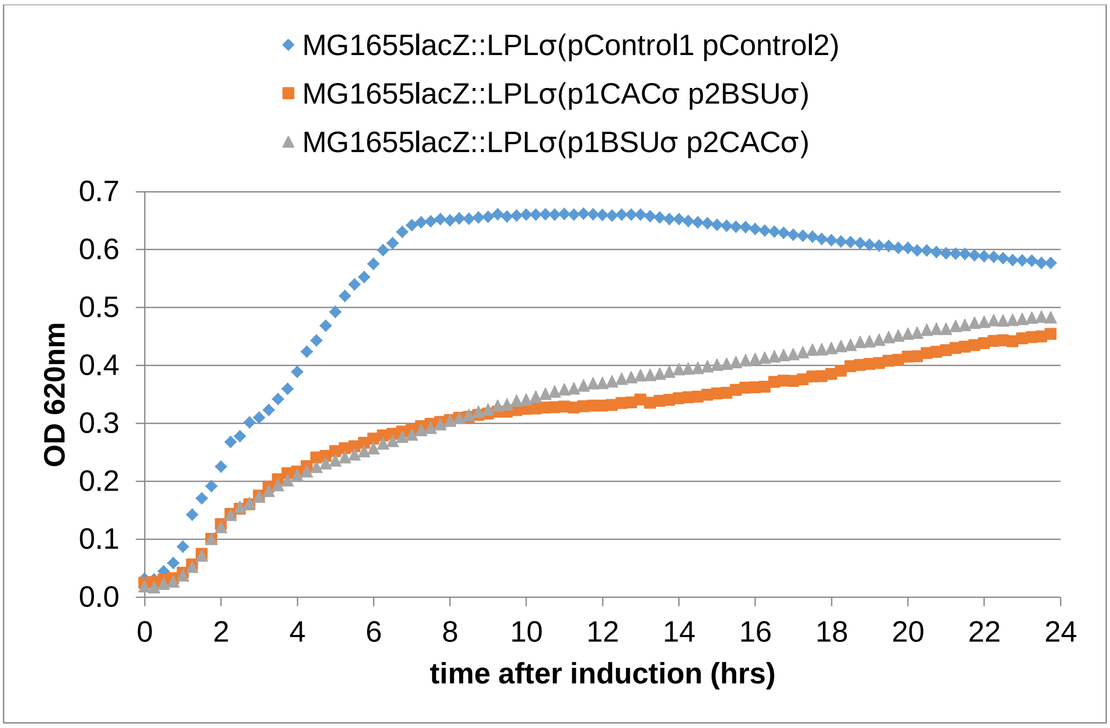

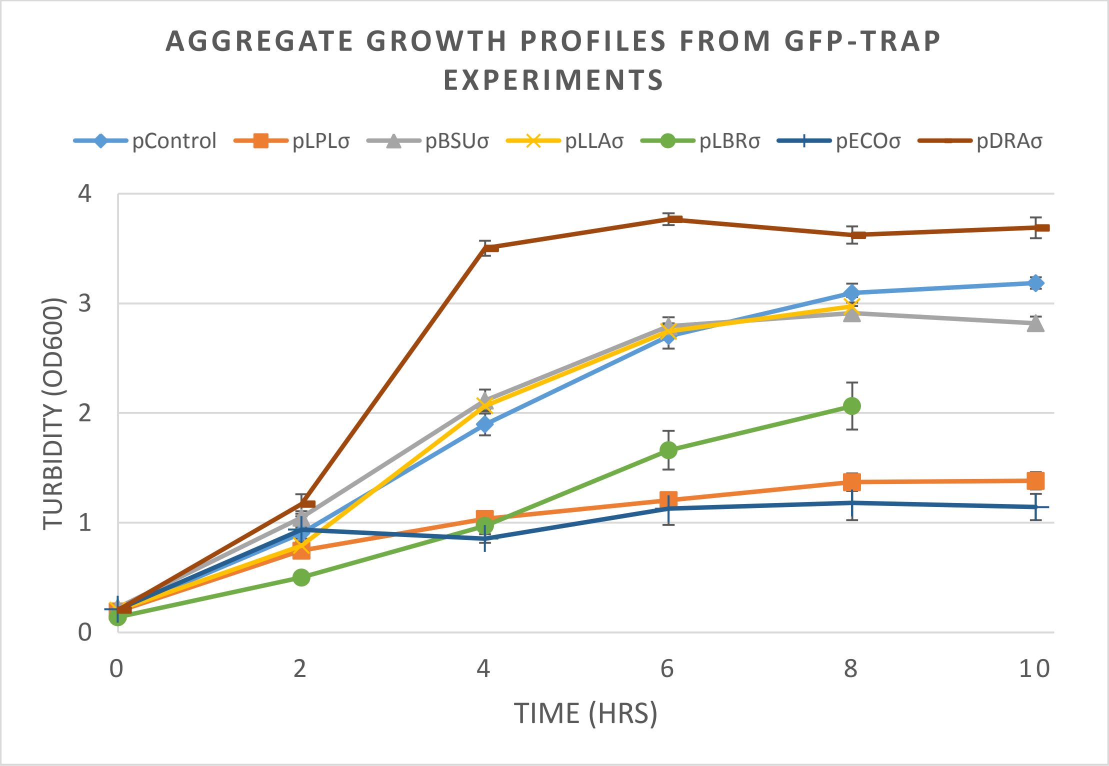

Supplementary Figure 7

Growth profiles of *E. coli* strains expressing combinations of heterologous sigma factors. (a) Expression of sigma factors was induced with 1 mM IPTG and the growth was followed via OD measurements in a 96-well plate reader. The profile of the control strain MG1655*lacZ*::*rpoD*(pControl, pControl2) is shown in blue; the profile of strain

MG1655*lacZ*::*rpoD*(pBSUσ, pCACσ-2) expressing *sigA* of *B. subtilis* from a low copy plasmid (pBSUσ) and *sigA* of *C. acetobutylicum* from a high copy plasmid (pCACσ-2) is shown in red; the profile of strain MG1655*lacZ*::*rpoD*(pCACσ, pBSUσ-2) expressing *sigA* of *C. acetobutylicum* from a low copy plasmid (pCACσ) and *sigA* of *B. subtilis* from a high copy plasmid (pBSUσ-2) is shown in green. Cultivations in 96-well plates usually do not reach as high cell densities as observed in larger flask cultures. (b) Aggregate growth profiles of the sigma factor expression strains from experiments with various GFP-trap libraries ($4 \leq n \leq 26$). pLLAσ represents the *Lactococcus lactis rpoD* expression strain; similarly, LBR is *L. brevis*, DRA is *D. radiodurans*, and ECO is *E. coli*. Error bars represent standard error.

Supplementary Figure 8

Random sample captures of pre-normalized data (read density) aligned by Integrated Genome Viewer against *E. coli* MG1655 genome map. All tracks are autoscaled to maximize height of tallest peak in track window of 6 kb and reads are not normalized. Dark Blue: Wild Type MG1655 plus strand, Light Blue: MG1655 *lacZ*::*rpoD* plus strand, Dark Red: Wild Type MG1655 minus strand, Light Red: MG1655 *lacZ*::*rpoD* minus strand. Gene images are taken from ecocyc.org[1]. The 6 kb regions selected by excel random number function [=rand()*4635000].

Region #1 - 759978-765978

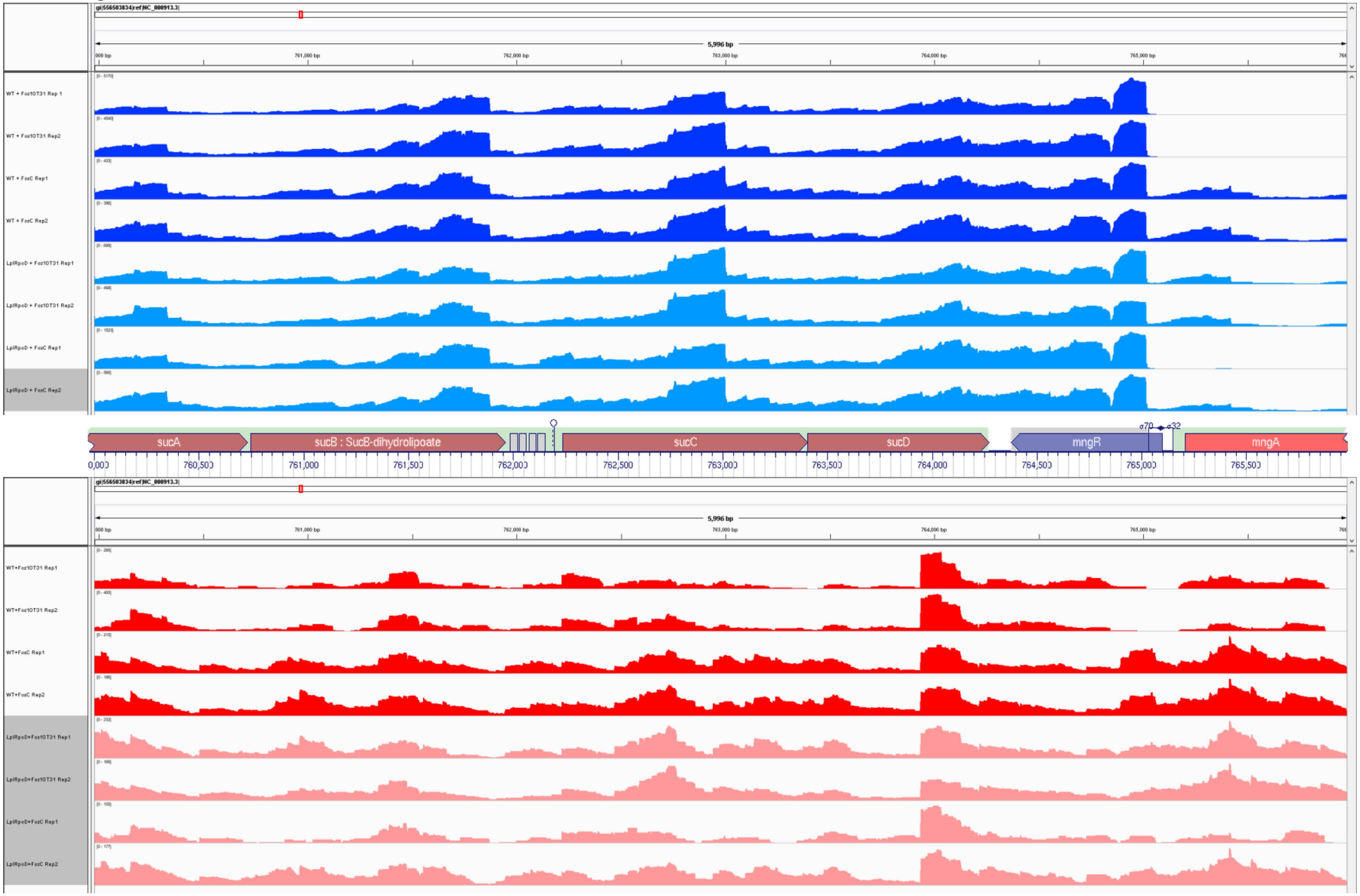

Region #2 - 855015-861015

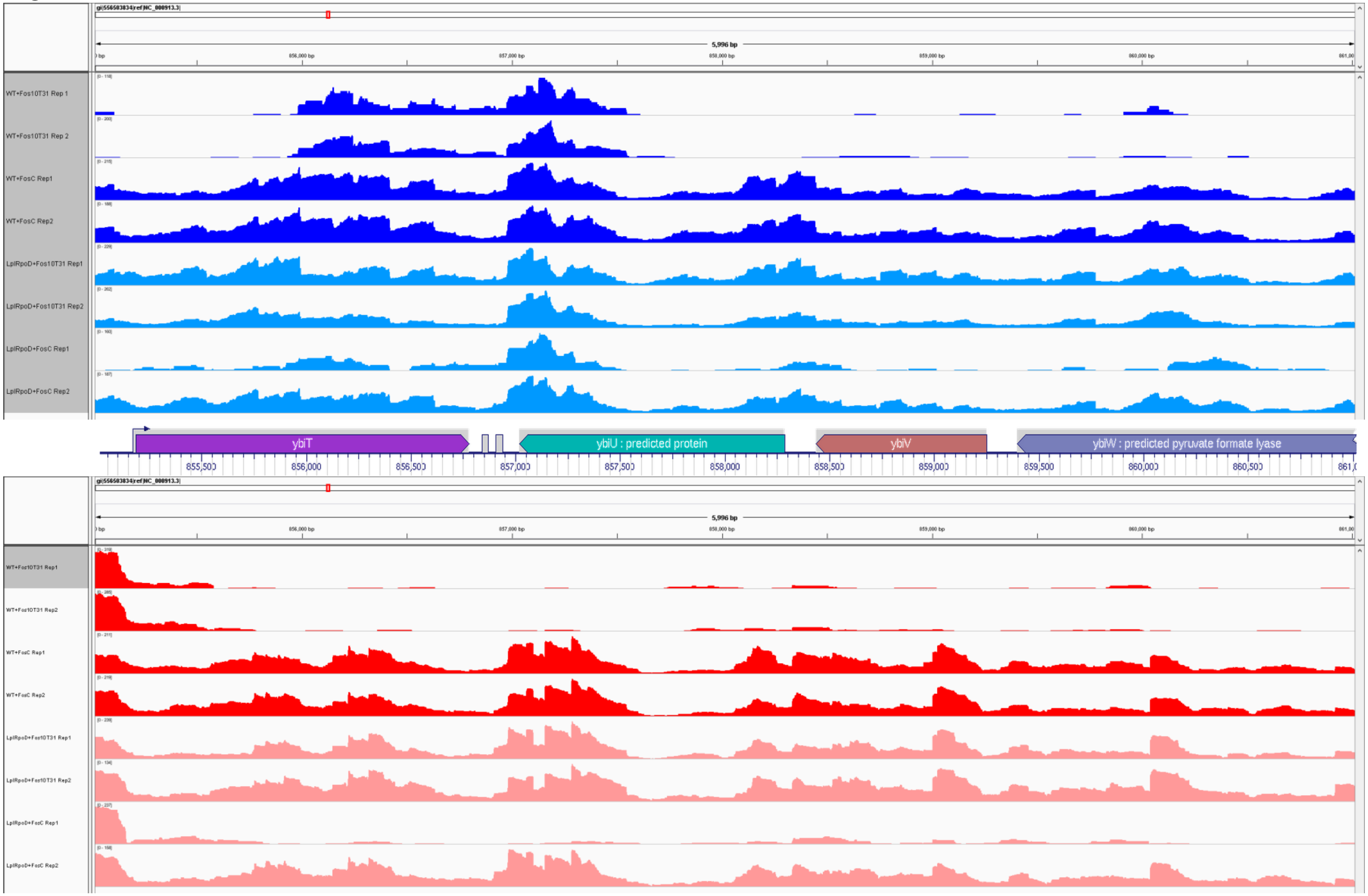

Region #3 - 1714250-1720250

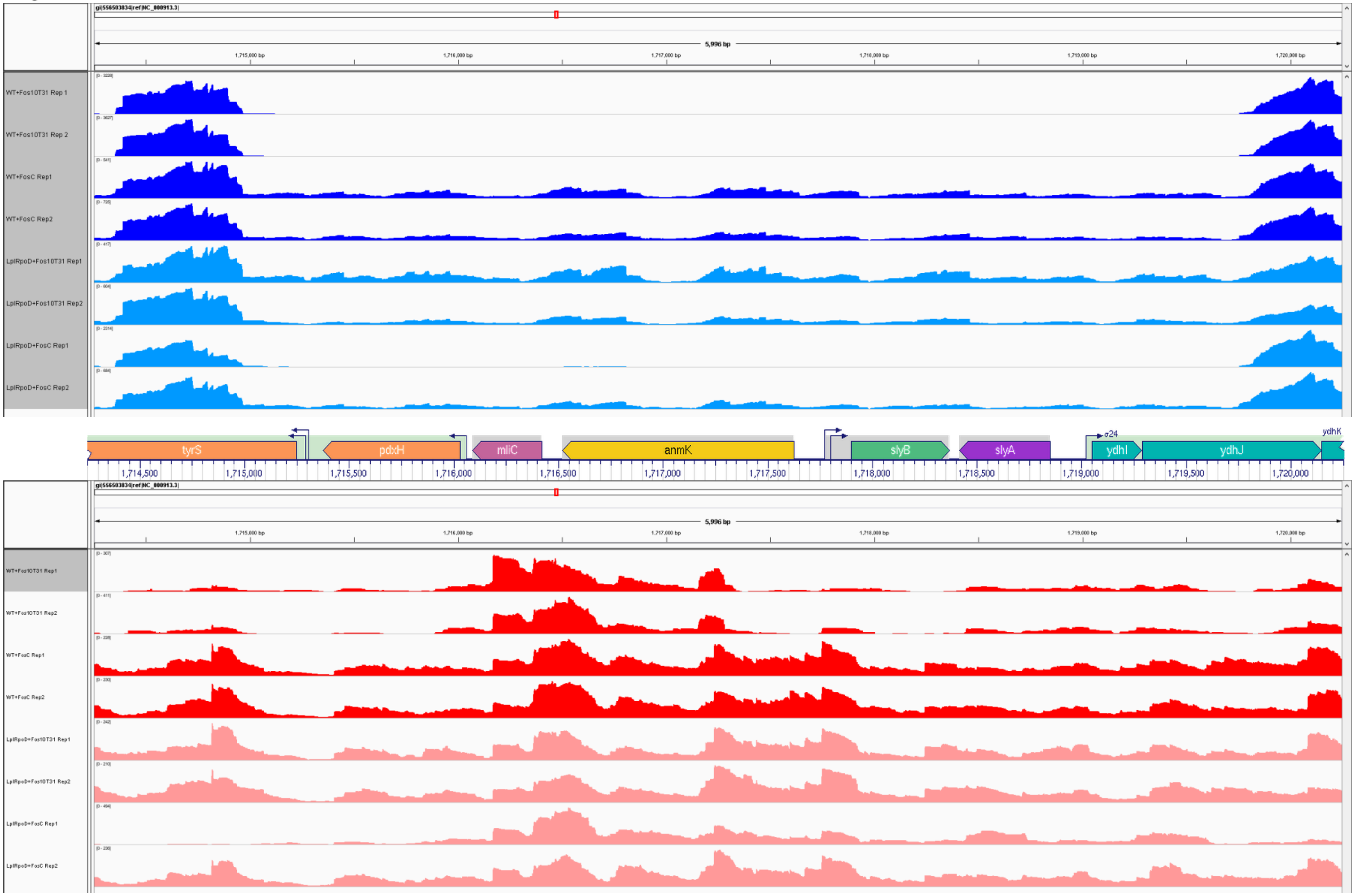

Region #4 - 1755127-1761127

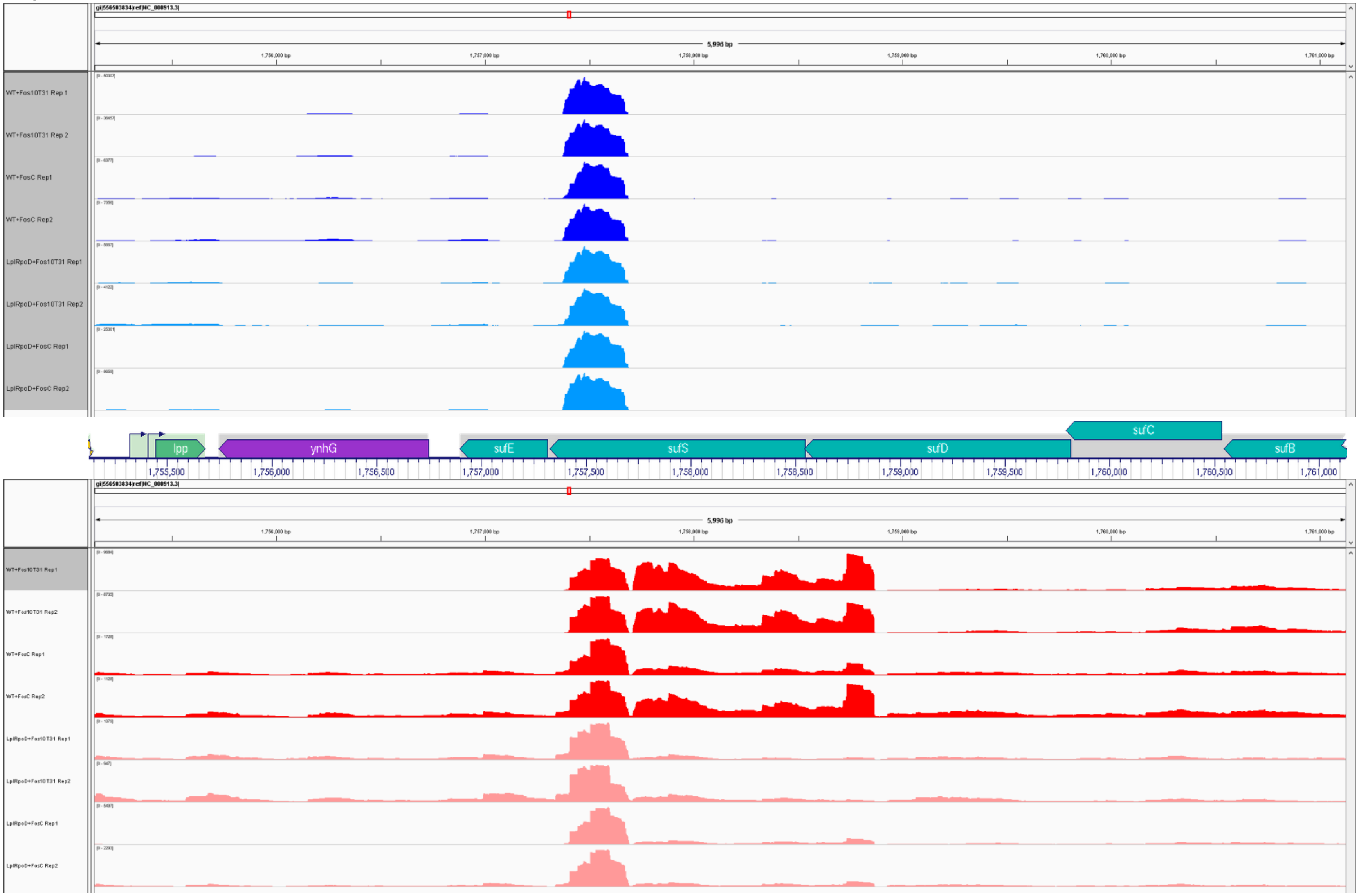

Region #5 - 1817479-1823479

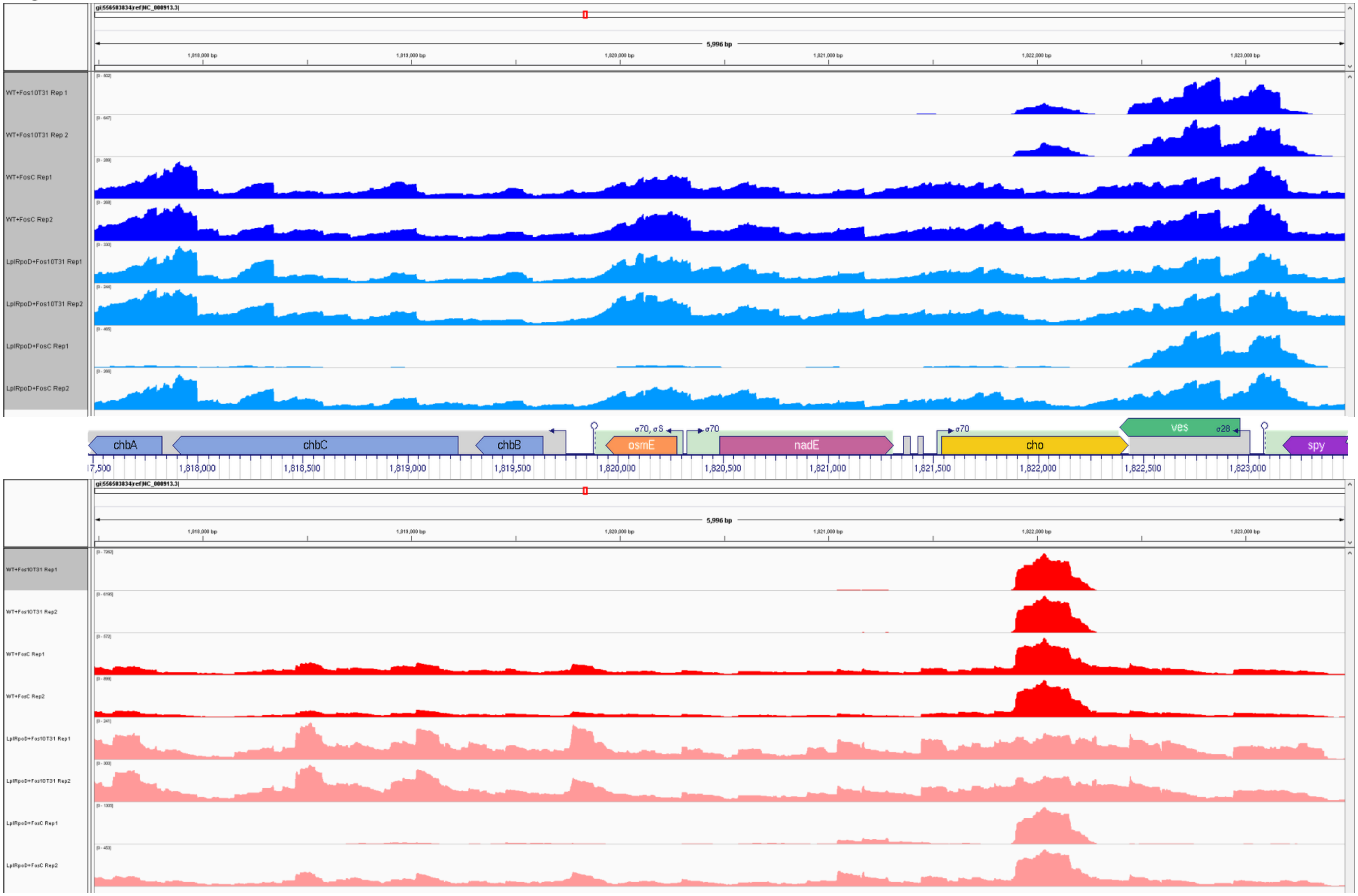

Region #6 - 1824547-1830547

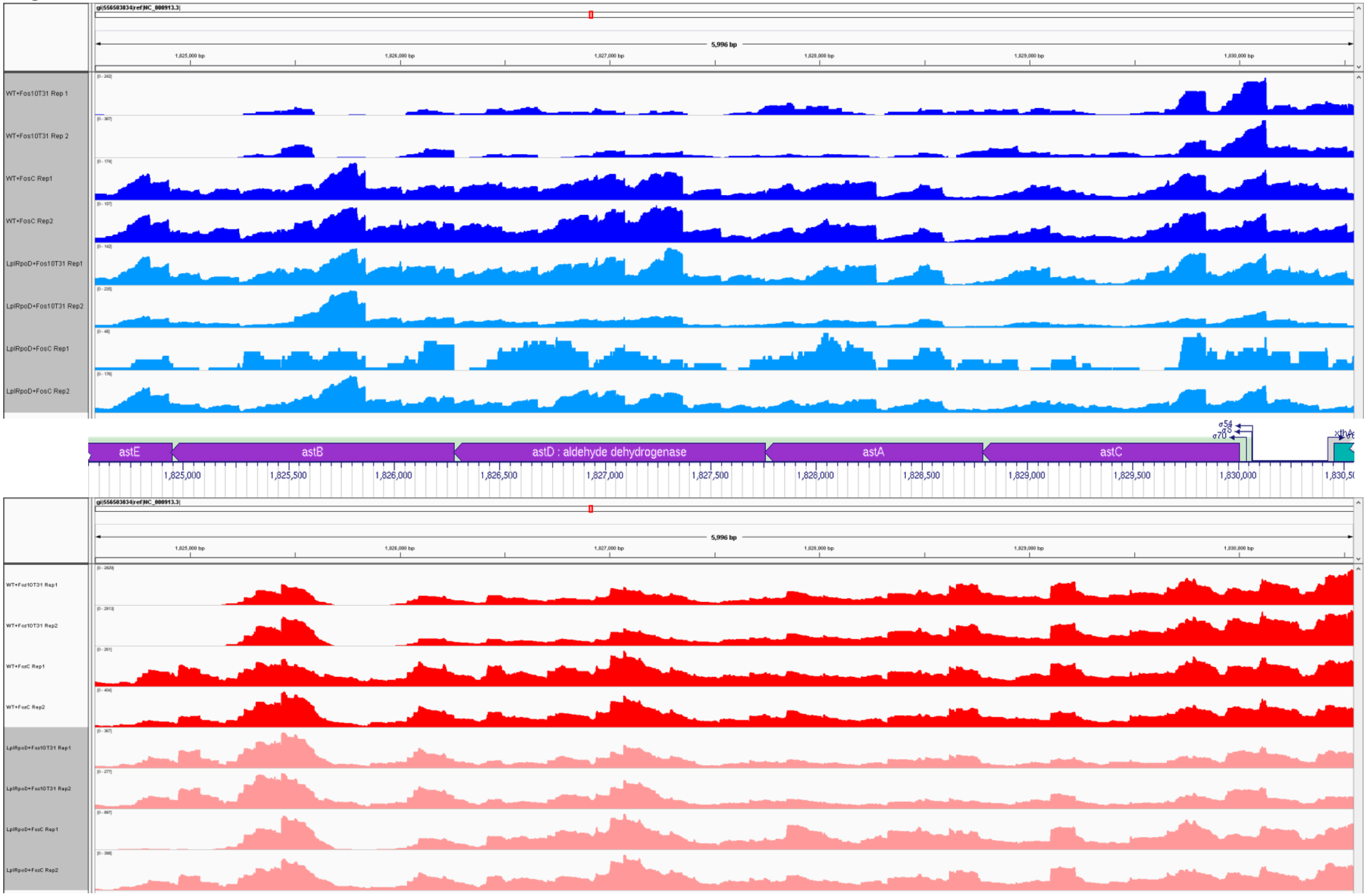

Region #7 - 2096514-2102514

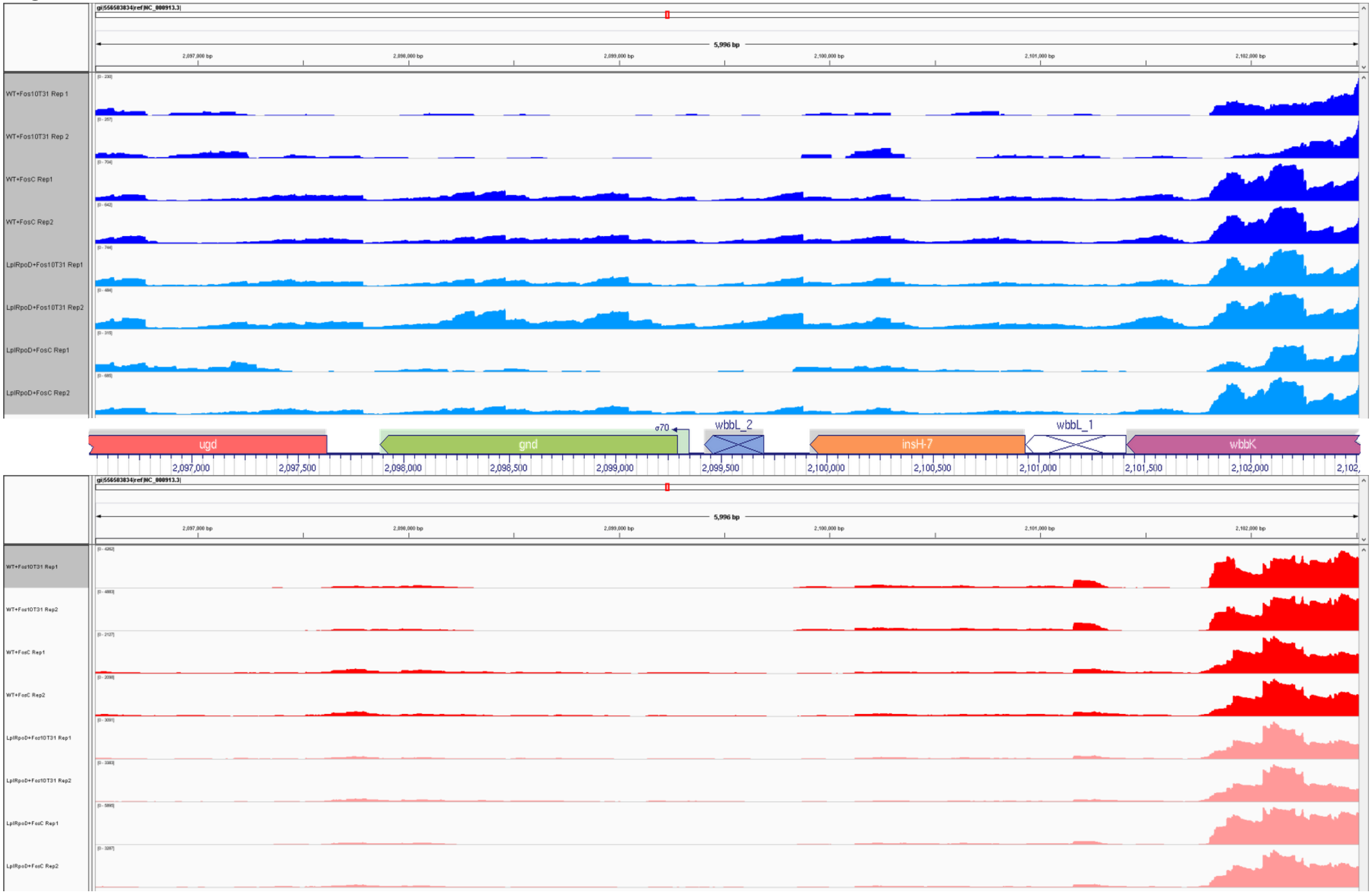

Region #8 - 2525839-2531839

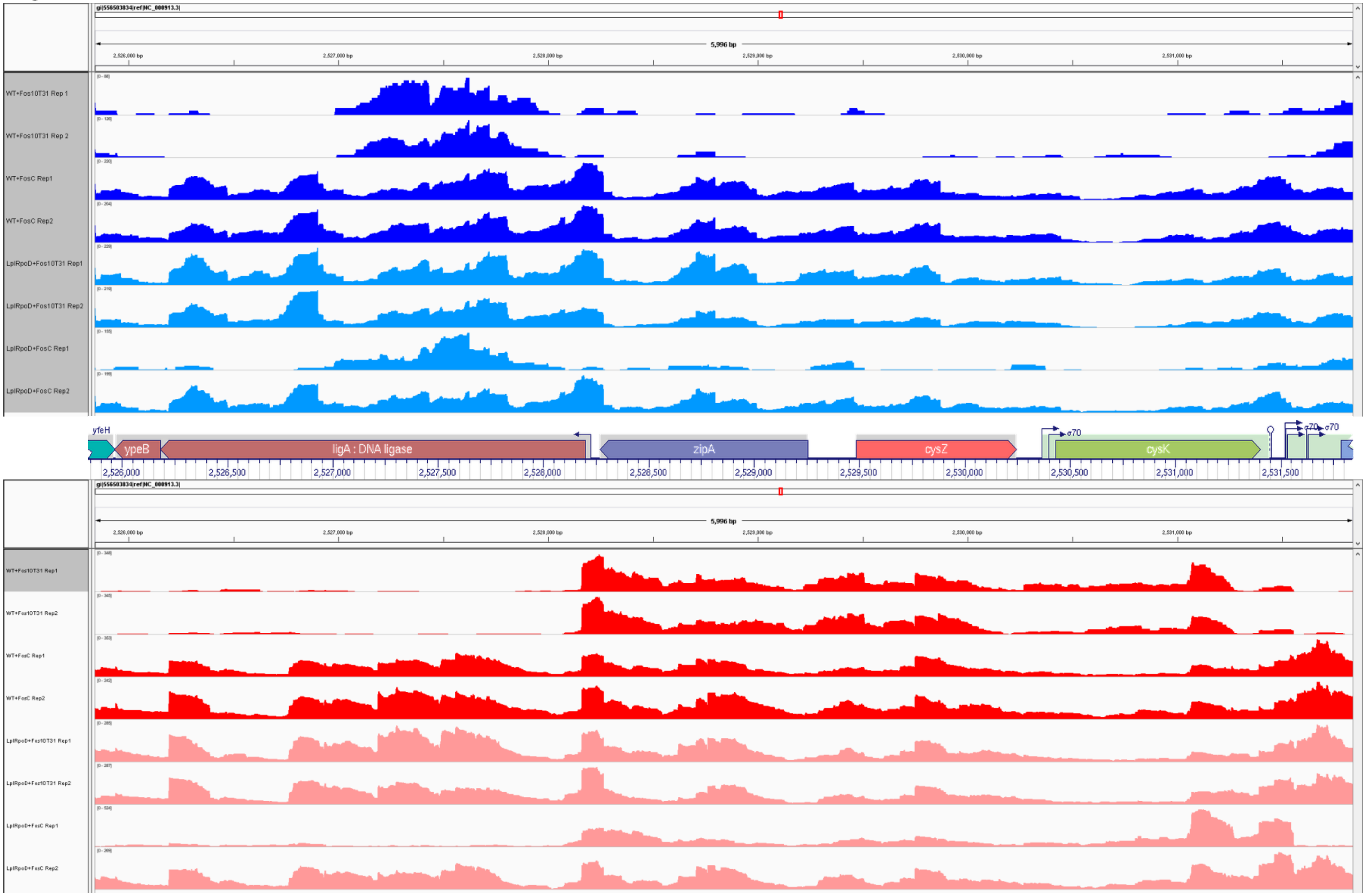

Region #9 - 2743383-2749383

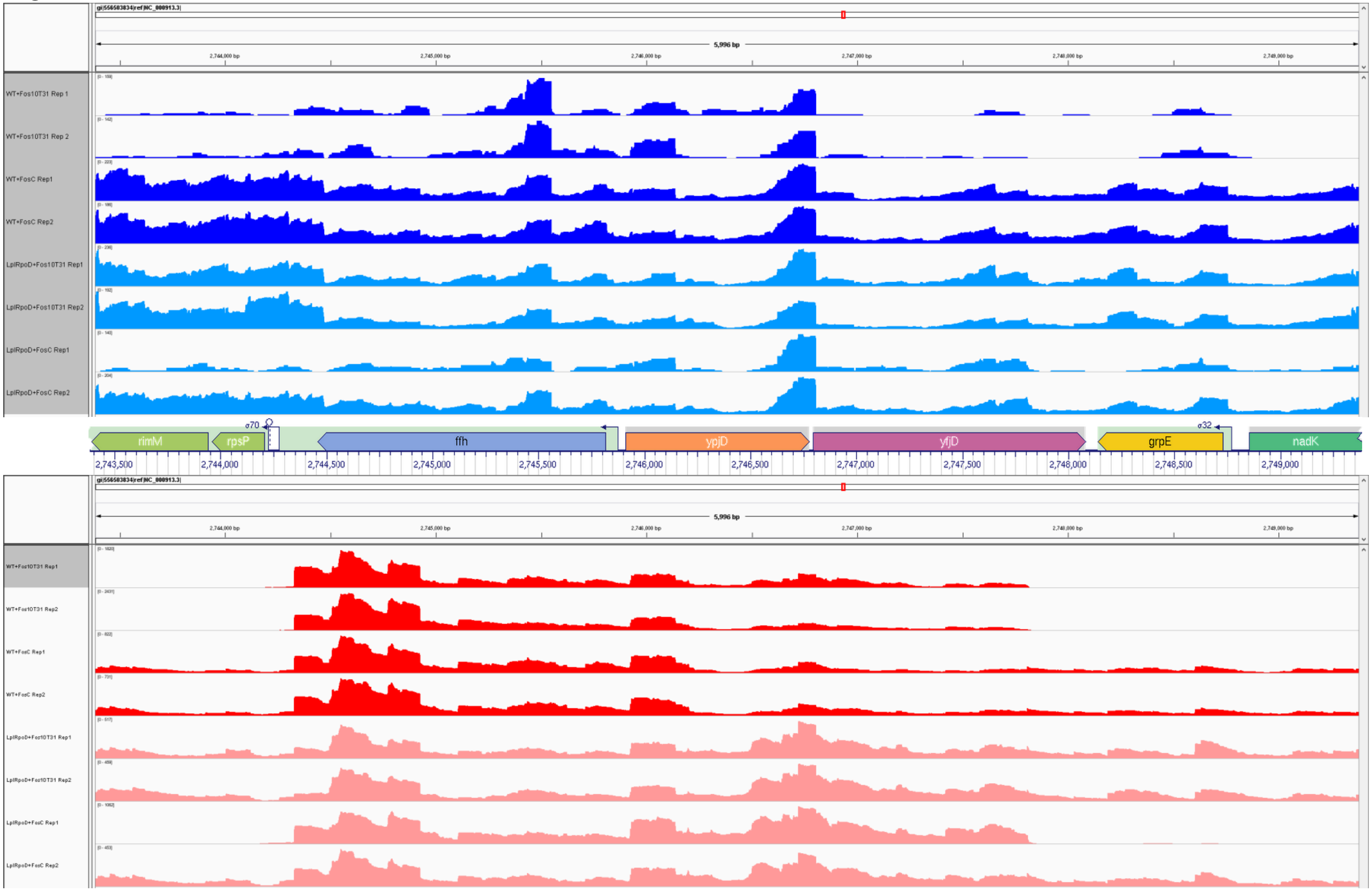

Region #10 - 3805911-3811911

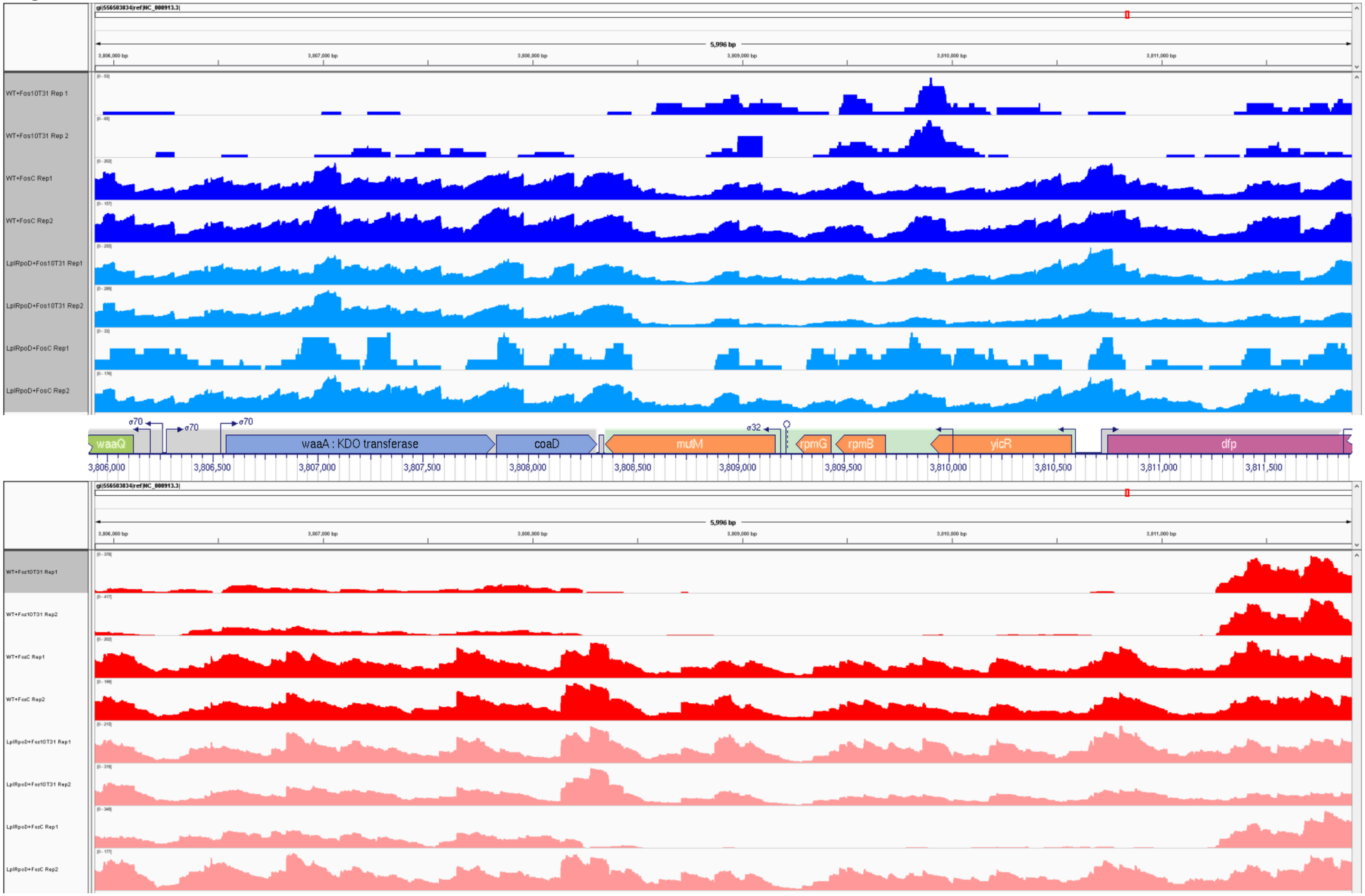

Region #11 - 4395694-4401694

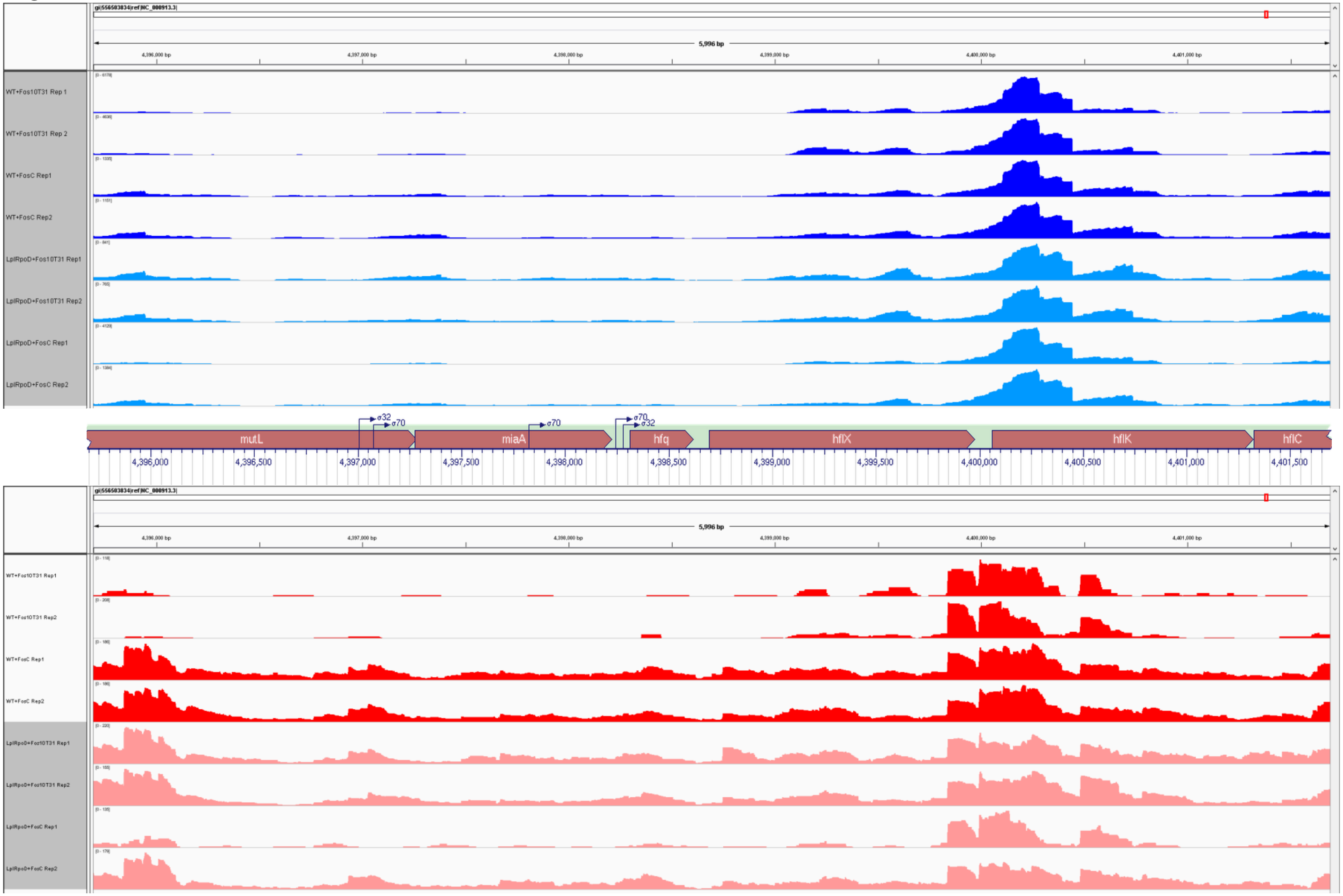

Supplementary Tables

Supplementary Table 1 Reported ethanol tolerant *E. coli* strains from the literature and their characteristics to benchmark the improvements observed for expression of fosmid Fos10T31.

Strain	Assay	Fold increase	Genetic modification	Reference
Fos10T31	Exposure of 24 and 48 hours in 7% ethanol	5- to 14-fold	Expression of *L. plantarum* locus 3,176,475…3,206,327	This study
∆slt / ∆afi / murB	Exposure of 8 hours in 7% ethanol	1.5- to 1.6-fold	Knockout & overexpression (plasmid based)	[2]
GroESL	Exposure of 24 hours in 6% ethanol	9-fold	Overexpression (plasmid based)	[3]
CoGel strains	Exposure of 24 and 48 hours in 4% ethanol	1.3- to 2.6-fold	Overexpression (plasmid & fosmid & co-overexpression)	[4]

Supplementary Table 2 Strains used in this study.

Strain	genotype	Source
E. coli MG1655	wild-type (E. coli K12 substrain MG1655)	CGSC
E. coli NEB10-beta	*araD139 ∆(ara,leu)7697 fhuA lacX74 galK16 galE15 mcrA f80d(lacZ∆M15)recA1 relA1 endA1 nupG rpsL rph spoT1∆(mrr-hsdRMS-mcrBC)*	NEB
E. coli NEB 5-alpha	*fhuA2∆(argF-lacZ)U169 phoA glnV44 Φ80∆ (lacZ)M15 gyrA96 recA1 relA1 endA1 thi-1 hsdR17*	NEB
E. coli NEB 5-alpha F’I^q	F´ *proA$^+$B$^+$ lacI^q ∆(lacZ)*M15 *zzf::Tn10* (Tet^R) */ fhuA2∆(argF-lacZ)U169 phoA glnV44 Φ80∆(lacZ)*M15 *gyrA96 recA1 relA1 endA1 thi-1 hsdR17*	NEB
E. coli One Shot®ccdB Survival^{TM}	F- mcrA Δ(mrr-hsdRMS-mcrBC) Φ80lacZΔM15 ΔlacX74 recA1 araΔ139 Δ(ara-leu)7697 galU galK rpsL (StrR) endA1 nupG fhuA::IS2	Invitrogen
E. coli One Shot ®TOP10	F- mcrA (mrr-hsdRMS-mcrBC) 80lacZM15 lacX74 recA1 ara139 (ara-leu)7697 galU galK rpsL (Str^R) endA1 nupG	Invitrogen
E. coli Epi300^{TM}-T1^R	*F$^-$ mcrA Δ(mrr-hsdRMS-mcrBC) Φ80dlacZΔM15 ΔlacX74 recA1 endA1 araD139 Δ(ara, leu)7697 galU galK λ$^-$ rpsL (Str^R) nupG trfA tonA*	Epicentre
Lactobacillus plantarum	wild-type (strain WCFS1)	ATCC
Bacillus subtilis	isolated gDNA from ATCC	ATCC
Clostridium acetobutylicum	wild-type (ATCC 824)	ATCC
E. coli MG1655*lacZ*::*rpoD*	*L. plantarum rpoD* knock-in *E. coli* strain	this study

ATCC: American Type Culture Collection (ATCC), Manassas, VA; **CGSC**: *E. coli* Genetic Stock Center, Yale University, New Haven, CT; **NEB**: New England Biolabs, Ipswich, MA; **Invitrogen**: Invitrogen, Carlsbad, CA; **Epicentre**: Epicentre, Madison, WI

Supplementary Table 3 Plasmids used in this study

Plasmids	Comments	Antibiotic resistance	Source
pUC19	expression plasmid (colE ori)	Amp^R	NEB
pACYCDuet™-1	expression plasmid carrying *lacI*, Cm^R and a p15A ori	Cm^R	Novagen
pCR®8/GW/TOPO®	entry plasmid for TOPO-TA cloning, containing attL sites	Spe^R	Invitrogen
pDEST™14	destination plasmid with LR-cassette (attR sites, colE ori)	Amp^R	Invitrogen
pDEST™40	destination plasmid with LR-cassette (attR sites, colE ori)	Amp^R	Invitrogen
pENTR™-gus	control entry plasmid carrying a promoterless *gus* from *Arabidopsis thaliana* coding for β-glucuronidase	Kan^R	Invitrogen
pLenti7.3/V5-GW/lacZ	expression plasmid carrying emGFP	Amp^R	Invitrogen
pCC1Fos™	CopyControl™ fosmid construction vector	Cm^R	Epicentre
pKD4	contains FRT-Kan^R-FRT cassette	Kan^R	CGSC
pKD20	helper plasmid 1 – λ Red system	Amp^R	CGSC
pCP20	helper plasmid 2 – Flippase	Amp^R	CGSC
pUC-kan	pUC19 with FRT-Kan^R-FRT cassette	Amp^R	this study
pUC-rpoD-kan	pUC19 with *L. plantarum rpoD* and FRT-Kan^R-FRT cassette	Amp^R	this study
pUC-GFP	GFP control plasmid	Amp^R	this study
pUC-LR-GFP	GFP-trap destination plasmid carrying *lac* promoter	Amp^R	this study
pLR-GFP	GFP-trap destination plasmid without *lac* promoter	Amp^R	this study
pACYC-LR	destination plasmid with LR-cassette (attR sites, p15A ori)	Cm^R	this study
pUC-Lpl-rpoD	*L. plantarum rpoD* construction plasmid # 1	Amp^R	this study
pCR®8-Lpl-rpoD	*L. plantarum rpoD* construction plasmid # 2	Sp^R	this study
pLPLσ	*L. plantarum rpoD* expression plasmid (p15A ori)	Cm^R	this study
pLPLσ-2	*L. plantarum rpoD* expression plasmid (colE ori)	Amp^R	this study
pUC-Lpl-rpoN	*L. plantarum rpoN* construction plasmid # 1	Amp^R	this study
pCR®8-Lpl-rpoN	*L. plantarum rpoN* construction plasmid # 2	Sp^R	this study
pLPL54	*L. plantarum rpoN* expression plasmid (p15A ori)	Cm^R	this study
pUC-Eco-rpoD	*E. coli rpoD* construction plasmid # 1	Amp^R	this study
pCR®8-Eco-rpoD	*E. coli rpoD* construction plasmid # 2	Sp^R	this study
pECOσ	*E. coli rpoD* expression plasmid (p15A ori)	Cm^R	this study
pUC-Bsu-sigA	*B. subtilis sigA* construction plasmid # 1	Amp^R	this study
pCR®8-Bsu-sigA	*B. subtilis sigA* construction plasmid # 2	Sp^R	this study
pBSUσ	*B. subtilis sigA* expression plasmid (p15A ori)	Cm^R	this study

pBSUσ-2	*B. subtilis sigA* expression plasmid (colE ori)	Amp^R	this study
pUC-Cac-sigA	*C. acetobutylicum sigA* construction plasmid # 1	Amp^R	this study
pCR®8-Cac-sigA	*C. acetobutylicum sigA* construction plasmid # 2	Sp^R	this study
pCACσ	*C. acetobutylicum sigA* expression plasmid (p15A ori)	Cm^R	this study
pCACσ-2	*C. acetobutylicum sigA* expression plasmid (colE ori)	Amp^R	this study
pControl	control plasmid (p15A ori)	Cm^R	this study
pControl2	control plasmid (colE ori)	Amp^R	this study
FosC	control fosmid	Cm^R	this study
Fos10T31	isolated fosmid	Cm^R	this study

CGSC: *E. coli* Genetic Stock Center, Yale University, New Haven, CT; **Novagen**: Novagen®, Merk KGaA, Germany; **NEB**: New England Biolabs, Ipswich, MA; **Invitrogen**: Invitrogen, Carlsbad, CA; **Epicentre**: Epicentre, Madison, WI

Supplementary Table 4 Oligonucleotides used in this study

Name	Sequence	Restriction sites
DEST-for	CCCACTGCTTACTGGCTTATCGAA	-
DEST-rev	TGATGATGACCGGTACGGGTAGAA	-
DUET-for	TTATAAACCAGGCGTTTAAGGGCACCAATA	-
DUET-rev	GATATCTAATGCAGGAGTCGCATAAGGGAG	-
Lpl-rpoD-for	GCGGTACCGCATACTAAATAGGAG	*Kpn*I
Lpl-rpoD-rev	CGAGCTCCATCTGGTGATTATTCC	*Sac*I
Lpl-rpoN-for	ATTATCCTGCAGGAGGACATTTATGGCACTGGGACCAGGA	*Sbf*I
Lpl-rpoN-rev	AGCATTGTAGAGTGGTACCTGGTCCAAATGGCGCGTTAAA	*Kpn*I
Eco-rpoD-for	ATGGTACCATAGCGTAGGAGGAATTTATGTTAGTCAGTATT	*Kpn*I
Eco-rpoD-rev	ATACTAGTACTTGAAGAGCTCCCGTACAATAACCACTTTAT	*Sac*I
Bsu-sigA-for	ATGGTACCATAGAATTCGTTGCAAGCTTT	*Kpn*I
Bsu-sigA-rev	GCCGAGCTCAAGGCATATTATCCATATATCT	*Sac*I
Cac-sigA-for	CGGGTACCATAGAATAACGGAAGGAGGTTT	*Kpn*I
Cac-sigA-rev	GCCGAGCTCGGTACTCTTCCAATATATTTAGC	*Sac*I
pUC19-for	TACCGCACAGATGCGTAAGGAGAA	-
pUC19-rev	CGATTCATTAATGCAGCTGGCACG	-
GFP-for	GCATAGGATCCTTAAGAAGGAGAACCTAGCATGGTGAGCAAGGGCGA	*Bam*HI
GFP-rev	TCCATGACAGCAGGACCGAATTCAGGTACCTTACTTGTACAGCTCGT	*Eco*RI
LR-for	ATTCCAGAGTCGAGCTGAAGCTTACAAGTTTGTACAAAAAAGCTGAA	*Hind*III
LR-rev	GCCGTCGCATGCTTAATTAGTTACCACTTTGTACAAGAAAGCTGAAC	*Sph*I
FRT-Kan-for	AGAGCTCGATTGTGTAGGCTGGAGCTG	*Sac*I
FRT-Kan-rev	GGAATTCTGGTCCATATGAATATCCTCC	*Eco*RI
lacI-for	ATATCCCGCCGTTAACCACCATCA	-
lacZ-rev	ACAAACGGCGGATTGACCGTAATG	-
RT-rpoD-for	TCGTGTCGACTTGTTAACGGCTGA	-
RT-rpoD-rev	TCATCAAGTCCGAACCGTAAGCGT	-
qRT-rpoD-for	TCGTGTCGACTTGTTAACGGCTGA	-
qRT-rpoD-rev	GCTTCGGCTAATTCTTGCTTGGCA	-
qRT-hcaT-for	GCTGATGCTGGTGATGATTGGCTT	-
qRT-hcaT-rev	ACTTTGCCGTAATCAAGCGGGAAC	-
Fos-for	GGATGTGCTGCAAGGCGATTAAGTTGG	-
Fos-rev	CTCGTATGTTGTGTGGAATTGTGAGC	-

Supplementary Notes

Supplementary Note 1. Estimation of GFP expression from the promoter GFP-trap libraries (LPL^{lac}-trap & LPL-trap)

The promoter GFP-trap libraries were constructed from random sheared genomic fragments of wild-type *Lpl*. Thus, the resulting inserts represent all locations of the chromosome without any bias. We assume that the genomic fragments are inserted into the library vector in both orientations without any bias. Depending on if and which regulatory element is present, GFP can or cannot be expressed from members of the two promoter GFP-trap libraries. For the LPL^{lac}-trap library, transcription is initiated by the promoter of the *E. coli lac* operon (P_{lac}) located upstream of the library insert. Thus, a terminator present in the insert would stop transcription and abolish GFP expression. In the LPL-trap library, only fragments containing an *Lpl* promoter, if recognized by the host, can initiate transcription and thus expression of GFP. Thus, the GFP expression profile from each library depends on the probability that an insert contains terminator or promoter sequences and/or their combinations as well as the arrangement of these regulatory elements on each library insert.

To estimate these probabilities, a simulation was carried out in R using the Bioconductor package[5] and the *Lpl* chromosome (NCBI Ref-Seq accession code NC_004567). For our simulations, we sampled a random fragment of the chromosome from a normal distribution with an average size of 726 bp and a standard deviation of 100 bp. The selected random fragment was then inspected for regulatory elements. Simulations were performed assuming the standard biological paradigm that a transcriptional unit (TU) contains an upstream promoter and a downstream terminator and that these elements are located in the intergenic regions (IR's) between TU's. In order to account for polycistronic TU's, gene pairs with an intergenic region smaller than 70 bp were assigned to belong to the same TU[6]. First, we determined the fraction of fragments, which do not contain any regulatory elements, and which will be referred to as ORF-only inserts (**Supplementary Fig. 1**, case a). Such inserts are composed of DNA belonging to an open reading frame (ORF) and/or a non-coding DNA between genes in an operon or IR shorter than what would be required for a functional promoter

or terminator. The cutoff for such IR segments was chosen to be 40 bp or less. Our simulation yielded a fraction of about 27% for ORF-only inserts.

Next, we estimated the fraction of fragments containing a complete IR of at least 71 bp, which will be referred as IR-only inserts (**Supplementary Fig. 1**, cases g-j). These inserts are assumed to contain a promoter as well as a terminator. This fraction was estimated to be about 11%. The remaining fraction of 63% (100% – 27% ORF-only – 11% IR-only) contain parts of an ORF as well as an IR fragments large enough to contain a regulatory element (>40 bp) and will be referred as ORF-IR inserts (**Supplementary Fig. 1**, cases k-p). For these inserts, we assume that the probability of containing a promoter (**Supplementary Fig. 1,** cases k and l) or terminator (**Supplementary Fig. 1**, cases m-p) is equal. So, about 31% contain one such regulatory element. Due to the random insertion of the fragment, the orientation of the regulatory element (promoter or terminator) will impact the transcription process. Promoters were assumed to be unidirectional and therefore only promoters directed towards the *gfp* can initiate *gfp* transcription (**Supplementary Fig. 1**, case k). Thus, we assume that half of all inserts containing a promoter, which is the sum of IR-only and ½ of ORF-IR inserts (11% + 31%=42%), will contain a promoter in the correct orientation to initiate transcription of *gfp* (**Supplementary Fig. 1**, cases g, h and k). This fraction is thus estimated to be about 21%. Rho-independent terminators are also unidirectional due to their T-rich tail needed to dissociate the RNAP from the DNA template [7] (**Supplementary Fig. 1**, cases g, i, m and n). Nevertheless, terminator predictions in *Lpl* show that many genes possess multiple terminators, which can be located on the sense as well as the anti-sense strand leading to transcription termination in both directions (TransTermHP database[7]). Inspecting the TransTermHP prediction for such terminators, we found about 910 genes that possess terminators located on opposite strands. This accounts for about half of the predicted TU's in *Lpl* [8]. So, for our calculations, we assume that half of the terminators are active in both directions and can terminate transcription independent of orientation (**Supplementary Fig. 1**, cases h, j, o and p). For the other half, we assume orientation dependency, so that only half of these can terminate transcription upstream of *gfp* (**Supplementary Fig. 1**, cases g and m). Thus, ¾ of all terminator containing inserts, which is the sum of IR-only and ½ of ORF-IR inserts (11% + 31%=41%) will stop transcription and therefore abolish GFP expression in the LPL^{lac}-trap library (**Supplementary Fig. 1**, cases g, h, j, m, o and p). This fraction was thus

estimated to be about 32%. Therefore the maximum GFP expression observable in the LPL^{lac}-trap would be 68%.

In the LPL-trap library, only fragments containing an *Lpl* promoter in the correct orientation can lead to GFP expression, which was estimated to be about 21% (see above). These predictions are based on the ideal model of a TU containing only one upstream promoter and one downstream terminator. It is known that additional promoters can be present in polycistronic TU's and lead to expression of a subset of genes present in such a TU. De Hoon et. al [9] reports that about 20% of genes assigned to polycistronic TU's carry such an additional promoter in the closely related organism *B. subtilis*. Such promoters would be found in ORF-only inserts. So, we assume that about 2/10 of ORF-only inserts also carry a promoter, whereby half of them are orientated towards *gfp* (**Supplementary Fig. 1**, cases b and c). These inserts (about 3%) can also lead to GFP expression in the LPL-trap.

Next, we inspected the TransTermHP terminator prediction of *Lpl* more closely. Here, more than a quarter of all predicted terminators were found in intragenic instead of intergenic regions (about 1141 out of 4027 predicted terminators). Again, these terminators would be found in the ORF-only insert fraction. To account for such intragenic terminators we assume that ¼ of the ORF-only inserts exhibit one (**Supplementary Fig. 1**, cases d-f). Again, due to the orientation dependency of the terminators we assume that ¾ of these inserts would terminate transcription upstream of *gfp* (**Supplementary Fig. 1**, cases e and f) and estimated this fraction to be about 6% of all inserts. Taking these adjustments into account, we estimate that about 38% of all inserts exhibit a terminator capable of abolishing GFP expression. So, for the LPL^{lac}-trap library with transcription initiated from P_{lac}, we would expect a maximum of 62% GFP positive cells. Experimentally, we found that about 54% of the inserts in the LPL^{lac}-trap library express GFP (**Fig. 2a**). Recent findings [10, 11] report that expression of GFP also depends on the genetic context between the promoter and the ribosomal binding site (RBS), whereby a strong hairpin sequence in the 5'UTR could prevent the binding of the ribosome to the RBS thus preventing GFP translation [12]. Thus, we speculate that the difference between the observed and the estimated value is mostly due to such secondary structures in our library inserts preventing GFP expression.

For the LPL-trap library, taking the adjustment for intragenic promoters into account, we estimate that at least 24% of all inserts carry an *Lpl* promoter in the correct orientation to initiate GFP expression (**Supplementary Fig. 1**, cases b, g, h and k). A

recent report by Todt et al. [13] undertook a genome-wide prediction of RpoD dependent promoters in *Lpl*. In that report, 3874 sequences were found to be potential RpoD dependent promoters in the upstream regions of protein-encoding genes, indicating that the genome exhibits more promoter-like sequences than open reading frames (3196). Some of these could be promoters of non-coding small RNAs [14]. So, as an alternative estimation method, we repeated the simulation without the assumption of polycistronic TU's and estimated the probability that an insert contains an upstream IR of an ORF of at least 40 bp, which is also oriented towards *gfp*. This fraction was estimated to be about 25%, which is in agreement with our former adjusted prediction of 24% promoter containing inserts. Experimentally, we observe a maximum of 23.4% of inserts expressing GFP during the expression of *Lpl rpoD*, indicating that most, if not all, of *Lpl* promoters can be transcribed in the presence of *Lpl* RpoD.

Supplementary Note 2. Verification of *Lpl rpoD* expression via sqRT-PCR and qRT-PCR

Expression of *Lpl rpoD* mRNA was verified via semi-quantitative reverse transcription PCR (sqRT-PCR). Differential expression between single chromosomal and plasmid-based expression was quantified via quantitative reverse transcription PCR (qRT-PCR). Overnight cultures of *E. coli* strain MG1655*lacZ::rpoD*, MG1655 (wild-type) as well as MG1655(pLPLσ) were used to inoculate (2%) 100 ml LB with appropriate antibiotics. Growth was monitored via OD_{600}. Expression of *Lpl rpoD* was induced with 1mM IPTG at an OD_{600} between 0.3 and 0.5 and cell pellets for RNA extraction collected 1, 2, 4 and 7 hours after induction. RNA was extracted using the RNeasy Mini Kit (Qiagen, Hilden, Germany) and 2 µg of total RNA were reverse transcribed via the High Capacity cDNA Reverse Transcription Kit (Applied Biosystems, Foster City, CA) according to the manufacturer's instructions.

cDNA of RNA extracted from *E. coli* strain MG1655*lacZ::rpoD* as well as from MG1655 was used to perform sqRT-PCR. 20 ng of cDNA were used in a Phusion PCR

reaction (NEB) with *Lpl rpoD* specific primers (RT-rpoD primer pair, **Supplementary Table 4**). The resulting PCR products of about 650 bp were separated and visualized on a 1% ethidium bromide agarose gel (**Supplementary Fig. 2a**).

Differential expression of *Lpl rpoD* between plasmid-based (MG1655 (pLPLσ)) versus chromosomal-based (MG1655 *lacZ::rpoD*) expression was detected as fold change via qRT-PCR and is shown in the **Supplementary Fig. 2b**. qRT-PCR was performed with SYBR® Green PCR Master Mix (Applied Biosystems) on a iCycler fitted with a iQ5 Multicolor Real-Time PCR Detection System (BioRad, Hercules, CA) as described [15]. The housekeeping gene *hcaT* was chosen to normalize cycle threshold values. qRT-PCR primers are listed in **Supplementary Table 4**.

Supplementary Note 3. Accounting for transcription initiation from plasmid backbone

In order to account for possible transcription initiation originating from the plasmid backbone, synthetic DNA was used to create random insert GFP-trap plasmids. Using a random number generator giving each base an equal probability of being called, a synthetic 750 bp insert was designed and ordered as a gBlock (49.1% GC; Integrated DNA Technologies, Coralville, Iowa):

```
AAACGATATA ATGTAAGAGC GATCCCTCCT AAGGCACCAA GTACGTTATG CTCGGGAATT
ATGAAAACAT GGTTTTTTGG TATACGATGG CCCTATCTGC CAAAAGTAGG TCGTCTCAGA
TACATGCGGA CGGAGGCCCC GCAGCGCGTA AGGTCGGTGT GCGGGATTCA ACCTGACGGG
AAACCCAAAA GTCTGAGAGA TCCGGTAATA TGTAACTCAT ATTCCTCCAA AATGGACGGG
CGTAGATGCT GTTTCTTAGT TCCCAACAGT CCCTACTGCC CAACGTTTCA AGTAAATATA
GTCAGGCATC AAGAATTGAT CACCCCACGT CGGGGCTAGT AAGCCGTGTT CCTTGCCAGC
TACATACTAT CCAGCGCGCC GCATACAGTA CGCCCGTGGA GATCTATCAG CATAAATCAT
CACTTATGTT AAGCGAACAC ACCGATAAGT TCTGGGTAGA GAGAAGTTGG GCCGGCAAGG
TTTATGCCCC CTATCCCCGC ATATTTAGTC CCACGTACCT CCCGCATCCT CCCGGGGGGT
CTGAGGCATT CTCACGCTCA GTTGCGGTGA GCGAGTTAGG GTCCTCTAAG AGACTTTTGC
CATAATTAGC TTGAACAACT AGCGGGAATC CTAAACTTTG GGAAATAGAC CTACCTTGCT
ATCACGCGAT TTCTAGGAGC GCTACTACAA CTTGTACGTT GGAATCGGAC AAGTGGGAGC
CCTTGAATCA ATAAGTTAGA TATCCGGTGG
```

The insert sequence was run through a transcriptional terminator prediction tool (Arnold, http://rna.igmors.u-psud.fr/toolbox/arnold) and no terminator was predicted in either direction of the insert. The insert sequence was also run through a prokaryotic promoter prediction tool (BPROM,

http://linux1.softberry.com/berry.phtml?topic=bprom&group=programs &subgroup=gfindb) where two very weak promoter sequences were predicted in either direction (score of 2.33 arbitrary units). Therefore the random insert, cloned in either direction, is not predicted to initiate transcription strongly nor terminate transcription. It was subsequently cloned into the pLR-gfp plasmid in the same manner as all the genomic libraries (adenylated with *Taq* polymerase, cloned into pCR®8/GW/TOPO, and recombined via LR rxn into pLR-gfp). We inserted this random element in both directions into the pLR-gfp plasmid to create pRandF-TRAP and pRandR-TRAP and co-transformed with either the control pACYC-gus or pACYC-Lpl-RpoD plasmid in the NEB 10β strain (four strains total). We examined these combinations in the same manner in which we tested the meta/genomic gfp-trap libraries via flow cytometry. These strains showed very little to no gfp+ population (under 1%) and there was no difference between the control and *Lpl* RpoD expressing strain (**Supplementary Fig. 3b**). Thus, transcription from the plasmid backbone is not a source of GFP expression.

Supplementary Note 4. Chromosomal integration and expression of sigma factors as an alternative to plasmid expression

Plasmid-based expression imposes limitations associated with vector stability and maintenance, as well as vector-compatibility concerns when screening plasmid-based libraries. To overcome these limitations, *Lpl rpoD* was integrated into the *lac* locus of *E. coli* under the control of P_{lac} (expression was verified via reverse transcription PCR, **Supplementary Fig. 2a**). To investigate if the gene dosage from the chromosomal expression is sufficient for *Lpl*-promoter recognition observed with *Lpl rpoD* expression from pLPLσ, the knock-in strain was transformed with the sorted sublibrary sLPL-trap and its GFP profile recoded (**Supplementary Fig. 4b**). Increased GFP expression was observed after 2 hours reaching a maximum of 7.3% 7 hours post induction, thus verifying that expression from a single chromosomal copy of *Lpl rpoD* is sufficient to enhance transcription in *E. coli* from *Lpl* promoters. Taking the reduced background of

the sorted LPL-library into account (about 5.4%), about half of all *Lpl* promoters (~ 12.7%) are recognized with the reduced gene dosage of *Lpl rpoD*. To better compete for the RNAP with intrinsic sigma factors the gene dosage could be increased by using stronger promoters or by protein engineering approaches.

Supplementary Note 5. Expression of the *Lpl rpoN*, coding for the alternative *Lpl* RpoN, enables the recognition of more *Lpl* promoters in *E. coli*

Prokaryotes adjust to altered environmental conditions, like stress, through expression of stress-specific sigma factors. We tested if expression of an alternative sigma factor from *Lpl*, notably RpoN (one of the two annotated alternative sigma factors in *Lpl* coded by *rpoN*) would further increase *Lpl* promoter recognition in *E. coli*. The predicted regulon of RpoN consist of about 21 genes representing about 0.66% of all *Lpl* genes[16]. Thus, increased *Lpl* promoter recognition resulting from expressing *rpoN* is expected to be small. To potentially detect such a small change in promoter recognition, the background signal due to *Lpl* promoter recognition by the native *E. coli* RNAP was decreased by fluorescent activated cell sorting (FACS) of the LPL-trap (above). FACS was carried out by sorting library clones based on their GFP signal and collecting GFP negative events. After re-cultivation of the collected clones, the GFP profile of the new sublibrary was acquired to verify enrichment of GFP negative clones. The library sorting resulted in a reduced fraction, from 6.5% to about 1.1%, of library inserts carrying *Lpl* promoters recognized by the native *E. coli* RNAP (**Supplementary Fig. 4a**). This new sub-library, designated as sLPL-trap, was then transformed in tandem with pLPL_RpoN expressing the *Lpl rpoN* gene. The GFP profile (**Supplementary Fig. 4a**) shows a larger cell population expressing GFP throughout the culture than for the sorted sublibrary. As expected, the difference is very small due to the small RpoN regulon. While the difference in GFP positive clones after 7 hours is not statistically significant (*t*-test, p=0.089), increased promoter recognition was observed consistently over 5 trials. This

strengthens the proposed concept and demonstrates that even small regulons of alternative sigma factors may be expressed and screened for.

Supplementary Note 6. Cross recognition between sigma factor/library pairs

During our experiments to examine possible cross recognition, we observed additional phenomena worth discussing here. Cross recognition of *Lpl* promoters was not observed (**Figure 2a**) when expressing the major sigma factor of *Clostridium acetobutylicum* (*sigA,* pCACσ) with the LPL-trap. A Gram$^+$ firmicute, *C. acetobutylicum* is phylogenetically further away to *Lpl* than *B. subtilis* is to *Lpl*, and thus, lack of cross recognition could be attributed to a reduced capability of the *C. acetobutylicum* SigA to recognize *Lpl* promoters. In addition a GFP-trap library with gDNA from *C. acetobutylicum* showed a very high GFP-expression profile in the control strain (pControl) (see **Figure 2d**). This may be due to the small insert size of the library (~250bp) and *Cac*'s low GC content (30.9%) (**Table 1**). We did observe a significant increase in GFP+ cells when the CAC-trap library was cotransformed with either pLPLσ or pBSUσ (**Figure 2d**). Conversely, the DRA-trap library showed extremely low GFP expression in the control strain (~1%) and has a very high GC content (~67%). This library would be poorly screened in an unmodified *E. coli* strain.

Supplementary Note 7. Effect of dual sigma factor expression on promoter recognition

We hypothesized that by expressing multiple sigma factors in a single strain, this would increase the level of transcription initiated from the heterologous inserts of the GFP trap libraries. To investigate this, we tested the transcription levels of a mixed LPL-trap and

BSU-trap library in the presence of individual and dual sigma factor expression (in the same manner previously described, **Methods**). The wild type control strain as well as the chromosomally integrated *Lpl rpoD* sigma factor expressing strain (in a NEB10β background) was transformed with either the control plasmid (pControl) or the *Bsu sigA* containing plasmid pBSUσ. In each case, the strains expressing a sigma factor significantly increased GFP expression over control (**Supplementary Fig. 5**). While the dual expression strain had the highest average GFP+ population (~24%), it was not statistically significantly higher than the strains containing individually expressed sigma factors *Lpl rpoD* (~18%) or *Bsu sigA* (~21%). We believe this may be due to an overlap in the recognition of promoter sequences, thus multiple sigma factors may not lead to an additive increase in heterologous transcription initiation.

Supplementary Note 8. Effect of sigma factor expression on growth

In order to investigate if *E. coli* can tolerate the simultaneous expression of more than one heterologous sigma factor, the following three expression strains were constructed and their growth behavior during simultaneous expression of three heterologous sigma factors examined First, *E. coli* strain MG1655*lacZ::rpoD*, expressing *Lpl rpoD* chromosomally, was transformed with pBSUσ (low copy *B. subtilis sigA* expression plasmid) and pCACσ-2 (high copy *C. acetobutylicum sigA* expression plasmid) to generate MG1655*lacZ::rpoD*(pBSUσ, pCACσ-2). Second, MG1655*lacZ::rpoD* was transformed with pBSUσ-2 (high copy *B. subtilis sigA* expression plasmid) and pCACσ (low copy *C. acetobutylicum sigA* expression plasmid) to generate MG1655*lacZ::rpoD*(pCACσ, pBSUσ-2). Third, as a control strain, MG1655*lacZ::rpoD* was transformed with pControl (low copy control plasmid) and pControl2 (high copy control plasmid) generating MG1655*lacZ::rpoD*(pControl, pControl2). Overnight cultures of these three strains were used to inoculate a 96-well plate filled with 200 µl LB containing IPTG to induce sigma-factor expression, and growth was monitored via optical density at 620 nm with a Sunrise™ 96-well plate reader (Tecan, Männedorf, Switzerland). The growth profile of these three strains is shown in **Supplementary**

Figure 7a indicating that *E. coli* can tolerate the simultaneous expression of multiple sigma factors and is viable. The growth rates and final optical densities observed were lower than those observed for the control strain.

During the course of our investigation, it was observed that certain sigma factors had an effect on growth rate. **Supplementary Fig. 7b** shows an aggregate growth profile of all the sigma factor expressing strains from the GFP-trap experiments. It is of note that the pLPLσ and the pECOσ strain have severely inhibited growth, perhaps indicating strong recruitment of the RNAP. The *Lactobacillus brevis rpoD* expression strain (pLBRσ) also exhibits a retarded growth, but did not increase GFP^+ population when tested against multiple libraries (**Supplemental Fig. 6**), which indicates recruitment of the RNAP is a necessary but not sufficient condition for increased promoter recognition.

Supplementary References

1. Keseler, I.M. et al. EcoCyc: fusing model organism databases with systems biology. *Nucleic acids research* **41**, D605-612 (2013).
2. Goodarzi, H. et al. Regulatory and metabolic rewiring during laboratory evolution of ethanol tolerance in E. coli. *Molecular Systems Biology* **6** (2010).
3. Zingaro, K.A. & Terry Papoutsakis, E. GroESL overexpression imparts Escherichia coli tolerance to i-, n-, and 2-butanol, 1,2,4-butanetriol and ethanol with complex and unpredictable patterns. *Metab Eng* (2012).
4. Nicolaou, S.A., Gaida, S.M. & Papoutsakis, E.T. Exploring the combinatorial genomic space in Escherichia coli for ethanol tolerance. *Biotechnol J* **7**, 1337-1345 (2012).
5. Gentleman, R.C. et al. Bioconductor: open software development for computational biology and bioinformatics. *Genome Biology* **5** (2004).
6. Paredes, C.J., Rigoutsos, I. & Papoutsakis, E.T. Transcriptional organization of the Clostridium acetobutylicum genome. *Nucleic Acids Research* **32**, 1973-1981 (2004).
7. Kingsford, C.L., Ayanbule, K. & Salzberg, S.L. Rapid, accurate, computational discovery of Rho-independent transcription terminators illuminates their relationship to DNA uptake. *Genome Biology* **8** (2007).
8. Mao, F.L., Dam, P., Chou, J., Olman, V. & Xu, Y. DOOR: a database for prokaryotic operons. *Nucleic Acids Research* **37**, D459-D463 (2009).
9. de Hoon, M.J.L., Makita, Y., Nakai, K. & Miyano, S. Prediction of transcriptional terminators in Bacillus subtilis and related species. *Plos Computational Biology* **1**, 212-221 (2005).
10. Lou, C., Stanton, B., Chen, Y.J., Munsky, B. & Voigt, C.A. Ribozyme-based insulator parts buffer synthetic circuits from genetic context. *Nat Biotechnol* **30**, 1137-1142 (2012).
11. Qi, L., Haurwitz, R.E., Shao, W., Doudna, J.A. & Arkin, A.P. RNA processing enables predictable programming of gene expression. *Nat Biotechnol* **30**, 1002-1006 (2012).
12. Carrier, T.A. & Keasling, J.D. Engineering mRNA stability in E. coli by the addition of synthetic hairpins using a 5' cassette system. *Biotechnol Bioeng* **55**, 577-580 (1997).
13. Todt, T.J. et al. Genome-Wide Prediction and Validation of Sigma70 Promoters in Lactobacillus plantarum WCFS1. *PLoS One* **7**, e45097 (2012).
14. Chen, Y., Indurthi, D.C., Jones, S.W. & Papoutsakis, E.T. Small RNAs in the Genus Clostridium. *Mbio* **2** (2011).
15. Jones, S.W. et al. The transcriptional program underlying the physiology of clostridial sporulation. *Genome Biology* **9** (2008).
16. Stevens, M.J.A., Molenaar, D., de Jong, A., De Vos, W.M. & Kleerebezem, M. sigma(54)-mediated control of the mannose phosphotransferase sytem in Lactobacillus plantarum impacts on carbohydrate metabolism. *Microbiology-Sgm* **156**, 695-707 (2010).
